# Prognostic model construction and immune microenvironment analysis of pyroptosis-related genes in hepatocellular carcinoma based on single-cell RNA sequencing

**DOI:** 10.3389/fimmu.2025.1595539

**Published:** 2025-08-21

**Authors:** Haoming Shen, Yizhi Peng, Qingqing Xie, Yuxi Ren, Junping Hu, Peifang Qin, Yuanxiong Chen, Hao Zeng, Yifan Sun

**Affiliations:** ^1^ Department of Clinical Laboratory, Hunan Cancer Hospital & The Affiliated Cancer Hospital of Xiangya School of Medicine, Central South University, Changsha, Hunan, China; ^2^ Department of Clinical Laboratory, Third Affiliated Hospital of Guangxi University of Chinese Medicine, Liuzhou, Guangxi, China; ^3^ Department of Clinical Laboratory, Eighth Affiliated Hospital of Guangxi Medical University, Guigang City People’s Hospital, Guigang, Guangxi, China

**Keywords:** hepatocellular carcinoma, biomarkers, pyroptosis, single-cell RNA sequencing, prognostic model

## Abstract

**Background:**

Hepatocellular carcinoma (HCC) prognosis continues to be challenging due to tumor heterogeneity and dynamic immunosuppressive microenvironments. Although pyroptosis plays a critical role in tumor-immune interactions, its prognostic significance in HCC at single-cell resolution has not been systematically investigated.

**Methods:**

We analyzed a publicly available single-cell RNA sequencing (scRNA-seq) data from 10 HCC tumors and paired adjacent tissue samples (60,496 cells) to elucidate pyroptosis-related gene (PRG) profiles. Differential expression and functional pathway analyses revealed PRG expression dynamics across cell subtypes. A LASSO-Cox prognostic model was developed using data from the liver hepatocellular carcinoma (LIHC) cohort of The Cancer Genome Atlas (TCGA) (n=365); the model was externally validated with International Cancer Genome Consortium (ICGC) datasets (n=231). Biological validation comprised reverse transcription quantitative polymerase chain reaction (RT-PCR) in HCC cell lines and immunohistochemical analysis of clinical specimens.

**Results:**

The scRNA-seq atlas identified 10 cellular clusters with enriched expression of 29 PRGs, primarily in natural killer cells, T lymphocytes, monocytes, and macrophages. The prognostic model developed in this study stratified patients into high-risk and low-risk categories based on eight significant genes, achieving area under the curve (AUC) values of 0.73, 0.65, and 0.69 for overall survival at one-year, two-year, and three-year intervals, respectively. Furthermore, external validation using data from the ICGC confirmed the prognostic model’s discriminative ability. Notably, high-risk patients demonstrated enhanced sensitivity to immunotherapy, as indicated by decreased tumor immune dysfunction and exclusion (TIDE) scores and increased expression of the immune checkpoints PD-1 and CTLA4.

**Conclusions:**

This study established a scRNA-seq-derived prognostic model based on PRGs, which offers insights into HCC immune landscape remodeling. The risk score and nomogram integrate tumor stages and pyroptosis-associated signatures, providing a clinical tool for personalized prognosis and therapeutic targeting.

## Introduction

Liver cancer remains a significant public health burden globally, ranking as the sixth most common cancer and the third leading cause of cancer death worldwide. Specifically, hepatocellular carcinoma (HCC) constitutes approximately 75%–85% of all primary liver malignancies (1). Notably, HCC is diagnosed in over 800,000 individuals annually, and exhibits mortality rates that parallel its incidence, with the five-year survival rate remaining below 20% despite therapeutic advances (2, 3). This dismal prognosis stems from delayed diagnosis, limited therapeutic efficacy, and the paucity of reliable molecular markers for early detection or recurrence prediction (4). While emerging immunotherapies targeting PD-1 checkpoint mechanisms show clinical promise (5), marked interpatient heterogeneity restricts treatment benefits to a subset of patients (5, 6). The immunosuppressive tumor microenvironment (TME) critically mediates both therapeutic resistance and immune escape mechanisms (7); this renders its modification a pivotal strategy for enhancing antitumor immunity and transforming HCC immunotherapy paradigms (8).

Pyroptosis is a lytic form of programmed cell death characterized by inflammatory activation that has emerged as a critical regulator of cancer progression and immune modulation (9, 10). Recent evidence suggests that pyroptosis-mediated inflammatory responses actively remodel the tumor immune microenvironment (TIME), thereby influencing immunotherapeutic efficacy (11, 12). In HCC, single-cell RNA sequencing(scRNA-seq) analyses have revealed pronounced enrichment of pyroptosis-related genes (PRGs) in malignant cells (13), while bulk transcriptomic studies have established PRG-based prognostic models (14–16). However, current prognostic models predominantly rely on clinicopathological parameters or individual molecular markers and lack integration with TME dynamics. Moreover, the precise spatial distribution and molecular interplay of PRGs in heterogeneous HCC tissue have not been systematically characterized at single-cell resolution. This knowledge gap limits our ability to exploit pyroptosis pathway modulators for precision oncology applications.

To address this gap, we systematically integrated single-cell and bulk transcriptomic data. ScRNA-seq data identified immune cell subsets enriched with PRGs, including NK cells, T cells, monocytes, and macrophages, establishing a biological basis for pyroptosis within the HCC immune microenvironment. Subsequently, using a LASSO-Cox regression algorithm, we developed and validated a biologically informed, this PRG-based framework provides a basis for prognostic model. This framework provides a basis for improved patient stratification and therapeutic target prioritization in HCC.

## Materials and methods

### Data collection and processing

The scRNA-seq dataset GSE149614 (17) was obtained from the Gene Expression Omnibus database; it included specimens from four locations in 10 HCC patients (10 primary tumors, 2 portal vein tumor thromboses, 1 metastatic lymph node, and 8 paracancerous tissue samples). The Illumina NovaSeq 6000 platform with the code GPL24676 was used for sequencing (*Homo sapiens*).

The R package Seurat (version 4.1.1) was used to perform quality control (18). Cells were excluded if they had fewer than 200 or more than 6,000 genes, as well as if they had more than 15% mitochondrial reads. Gene expression was normalized using Seurat’s LogNormalize method. After adjusting for average expression and dispersion, we identified highly variable genes in single cells. Principal component analysis (PCA) was performed on these genes, and important components were identified using the ElbowPlot method. The PCElbowPlot in Seurat highlighted five noteworthy components.

Data for 371 cases of liver hepatocellular carcinoma (LIHC) were acquired from the Cancer Genome Atlas (TCGA) GDC using the R package TCGAbiolinks (version 2.22.4) (19). This dataset included FPKM expression profiles, count matrices, survival data, and clinical information (371 cancerous vs. 50 noncancerous tissue samples). From these, 337 cases had comprehensive clinical and survival data (**Supplementary Table S1**). A validation set was obtained from the International Cancer Genome Consortium (ICGC) (20) with data for 231 patients with HCC (**Supplementary Table S1**).

In order to construct a well-conserved list of PRGs for further prognostic screening without selection bias, we extracted 85 PRGs from three independent resources, including GeneCards database (https://www.genecards.org/), Molecular Signatures Database (MSigDB, version 7.4, http://tardis.cgu.edu.tw/msignaturedb) (21) and our systematic literature review (15, 22, 23). Specifically, six genes (including GSDMD and CASP1) were identified from GeneCards with relatedness score more than 7 based on careful investigation. Meanwhile, 27 PRGs were extracted from MSigDB according to pyroptosis-related pathways. In addition, 70 PRGs were collected from more than 30 studies published in literature concerned with pyroptosis in cancer, especially HCC. For instance, TREM2 was selected for its immune evasion effect mediated by macrophages, while CHMP4B was chosen for its effect on pyroptosis execution by ESCRTs. Due to partial gene overlap among the three sources, we merged all obtained genes, removed duplicates, and ultimately identified 85 unique pyroptosis-related genes for subsequent analysis (**Supplementary Table S2**).

### Identification and functional enrichment analysis of pyroptosis marker genes

For our study, cells were clustered into ten groups using the FindClusters function in Seurat with the resolution set at 0.1. Each cell was then classified using the SingleR package (version 1.8.1) (24), referencing the HumanPrimaryCellAtlasData. It should be noted that for the cell population annotated as hepatocytes, no further subclustering was performed in this study. Methods designed to distinguish malignant from non-malignant cells based on inferred copy-number variations (CNVs), such as inferCNV, were not applied. Therefore, for the purpose of this analysis, all cells identified as hepatocytes were treated as a single entity. Differential gene expression analysis was performed using Seurat’s FindAllMarkers function, focusing on the intersections of PRGs. To quantify gene set activity, we applied the AUCell package (version 1.16.0) (25), classifying cells with an enrichment score above 0.13 as high scoring. The threshold of 0.13 was determined based on the bimodal distribution of AUCell scores across all cells, providing a data-driven, albeit simplified, method to delineate cells with high versus low pyroptosis-related gene set activity. These cells underwent further clustering, annotation, and marker-gene analysis. The cell cluster with high-scoring cluster exhibited enrichment in Gene Ontology (GO) analysis, while the differentially expressed genes within this cluster showed enrichment in Kyoto Encyclopedia of Genes and Genomes (KEGG) analysis, utilizing the clusterProfiler (R package v4.2.2) (26). Pseudotime trajectory analysis of these clusters was carried out with Monocle (version 2.22.0) (27) to unravel molecular mechanisms in HCC progression. The CellChat package (version 1.5.0) (28) helped explore intercellular communication within these clusters.

### Identification and functional enrichment analysis of pyroptosis key genes

We used the DESeq2 package (version 1.34.0) (29) to discern DEGs between normal and tumor tissue in the TCGA database, with |log2(FC)|>0 and FDR <0.05 as the screening criteria. The DEGs were then cross-referenced with pyroptosis marker genes to identify key genes. GO and KEGG functional enrichments for these key genes were conducted using “clusterProfiler” (26). Samples were clustered via ConsensusClusterPlus (version 1.58.0) (30), utilizing the partition around medoids (PAM) method. ConsensusClusterPlus output, along with gene expression heatmaps, Cumulative Distribution Function, and Delta Area Plot, was meticulously analyzed. A boxplot displayed the differential expression of key pyroptosis genes across various subtypes. Lastly, we extracted 16 immune cell types (**Supplementary Table S3**) and 13 immune-related gene sets from the literature (**Supplementary Table S4**). The ssGSEA method within the GSVA package (version 1.42.0) (31) was employed to examine their enrichment in tissue samples.

### Construction and validation of the prognostic risk model

For the construction of our risk model, we employed RNA-Seq datasets from TCGA-LIHC, which included 337 HCC tumor samples. The ICGC dataset, which comprised 231 HCC samples, served as the validation cohort to confirm the model’s effectiveness. Covariates with P < 0.3 in univariate Cox analysis were included in the multivariate model. This inclusive threshold follows statistical recommendations to retain potentially informative variables for multivariate adjustment. LASSO regression was used to improve the predictors, and multivariate Cox regression was used to create the risk score equation, which was calculated as the sum of the product of gene expression and the coefficient of each gene. The optimal cutoff for gene expression was determined using the survminer package (version 0.4.9). To assess the predictive accuracy of the model, we conducted a Kaplan–Meier survival analysis and time-dependent ROC curve analysis.

### Constructing the clinical prediction model

We utilized univariate Cox proportional hazards regression to evaluate the association of the risk score and clinical characteristics with overall survival (OS) in HCC patients. The variables found to be predictive in the initial analysis, in addition to important clinical variables, were included in a multivariable Cox proportional hazards model. From this model, we developed a nomogram to predict HCC prognosis, thereby offering a personalized, visual representation of risk. A calibration curve was created to evaluate the predictive accuracy of the nomogram by comparing the projected survival probabilities to the actual results.

### Dysfunction and exclusion of tumor immunity

The tumor immune dysfunction and exclusion (TIDE) framework (32) was used to forecast immune evasion strategies and evaluate the effectiveness of immune checkpoint inhibitors in cancerous tissue. We used the TIDE web tool to compute TIDE scores for individual tumor samples and analyzed the relationship between these scores and the risk scores. Additionally, we investigated the relationship of four immune checkpoints—PD-1, PD-L1, PD-L2, and CTLA4—with the risk score to further understand their prognostic implications.

### Immune landscape analysis

Immune cell infiltration differences between high- and low-risk HCC patient groups were analyzed using the CIBERSORT.R script available at CIBERSORTx (http://CIBERSORT.stanford.edu/) (33). Utilizing the LM22 signature matrix and 1,000 permutations, 22 immune cell subsets were characterized, including naive and memory B cells, plasma cells, various T cell types, resting and activated NK cells, monocytes, different macrophage phenotypes, resting and activated dendritic cells, mast cells, eosinophils, and neutrophils. The variations in immune cell infiltration were shown in box plots comparing the proportions of these 22 immune cell types in the tumor tissues of high- versus low-risk patients.

In investigating somatic mutations, the TCGA-LIHC dataset was statistically analyzed with the maftools package (version 2.10.5) (34). We procured Masked Copy Number Segment data for patients using the TCGAbiolinks package. The CNV profiles were determined by GISTIC 2.0 analysis (35), and the analysis results were visualized by maftools package (34).

### Drug sensitivity analysis

We obtained the gene expression profiles of 60 cell lines and the IC50 values for 24,360 drugs from the CellMiner database (https://discover.nci.nih.gov/cellminer) (36). After excluding any datasets with incomplete information, we analyzed the correlation between prognostic genes and the IC50 values of 62 selected drugs.

### Tumor immune dysfunction and exclusion

To predict immune escape mechanisms and assess the potential efficacy of immune checkpoint inhibitors (ICIs), we employed the Tumor Immune Dysfunction and Exclusion (TIDE) framework (32). For this analysis, FPKM expression data from the TCGA-LIHC cohort were submitted to the TIDE web portal (http://tide.dfci.harvard.edu/), which applies internal normalization based on reference z-scoring. It is important to clarify that a higher TIDE score predicts a greater potential for tumor immune evasion and thus a lower likelihood of benefiting from ICI therapy. Furthermore, this score is primarily derived from gene expression signatures associated with T cell dysfunction and exclusion, rather than the entire immune landscape. Using the calculated TIDE scores, we then examined the correlation with our prognostic risk score and the expression of four immune checkpoints (PD-1, PD-L1, PD-L2, and CTLA4).

### Reverse transcription quantitative polymerase chain reaction

We measured the mRNA expression levels of *BAX*, *CHMP4B*, *TREM2*, *CHMP3*, *GBP1*, *IRF1*, *CHMP2A*, and *MST1* in LX-2 liver cells and various HCC cell lines, including SNU-182 (Abiowell, AW-CCH045), HCC-LM3 (Abiowell, AW-CCH210), Hep3B (Abiowell, AW-CCH035), HepG2 (Abiowell, AW-CCH024), Huh-7 (Abiowell, AW-CCH089), and MHCC-97H (Abiowell, AW-CCH088). mRNA extraction was performed using the TRIzol method. Next, reverse transcription was conducted using a Mastercycler gradient thermal cycler (Eppendorf) with a kit from Beijing CwBio Technology Co. Ltd.; *GAPDH* was the reference gene. The cDNA thus obtained was amplified using PCR according to the instructions included in the mRNA amplification kit, using an ABI 7500 PCR system from Applied Biosystems (Thermo Fisher Scientific Inc.). The reverse transcription quantitative polymerase chain reaction data were analyzed with the ^ΔΔ^CT method. The specific primer sequences are listed in **Supplementary Table S5**.

### Immunohistochemistry

Fresh tissue samples from two HCC patients were obtained from the Hunan Cancer Hospital after approval from the hospital’s Ethics Committee (approval no. 2023-092). The following primary antibodies were used for immunohistochemistry: a polyclonal rabbit anti-CHMP2A (Proteintech, Cat# 10477-1-AP), a polyclonal rabbit anti-CHMP3 (also known as VPS24) (Proteintech, Cat# 15472-1-AP), a polyclonal rabbit anti-CHMP4B (ThermoFisher, Cat# PA5-100092), and a polyclonal rabbit anti-TREM2 (Thermo Fisher, Cat# PA5-116068). The detailed immunohistochemistry procedures were as previously described (37). The processed tissue sections were digitally scanned with an Aperio ImageScope scanner for analysis.

### Statistical analysis

R (version 4.1.3) was used for all data processing and analysis. When comparing two sets of continuous variables, the independent samples t-test was used to evaluate the statistical significance of variables that were normally distributed, whereas the Mann–Whitney U test was employed to examine discrepancies among variables that were not normally distributed. Categorical variables were compared and analyzed for statistical significance using the chi-square test or Fisher’s exact test. Survival analysis was conducted with the Survival software; Kaplan–Meier survival curves were displayed to show differences in survival, and the significance of survival time disparities between groups was assessed with the log-rank test. Cross curves were generated with timeROC (R package version 0.4) to evaluate the risk score’s accuracy in predicting prognosis by calculating the area under the curve (AUC). Univariate and multivariate Cox analyses were employed to identify independent prognostic factors. All the statistical tests were two tailed, with the significance level set at P < 0.05.

## Results

### Single-cell data reveal cellular heterogeneity in HCC

**Figure 1:**
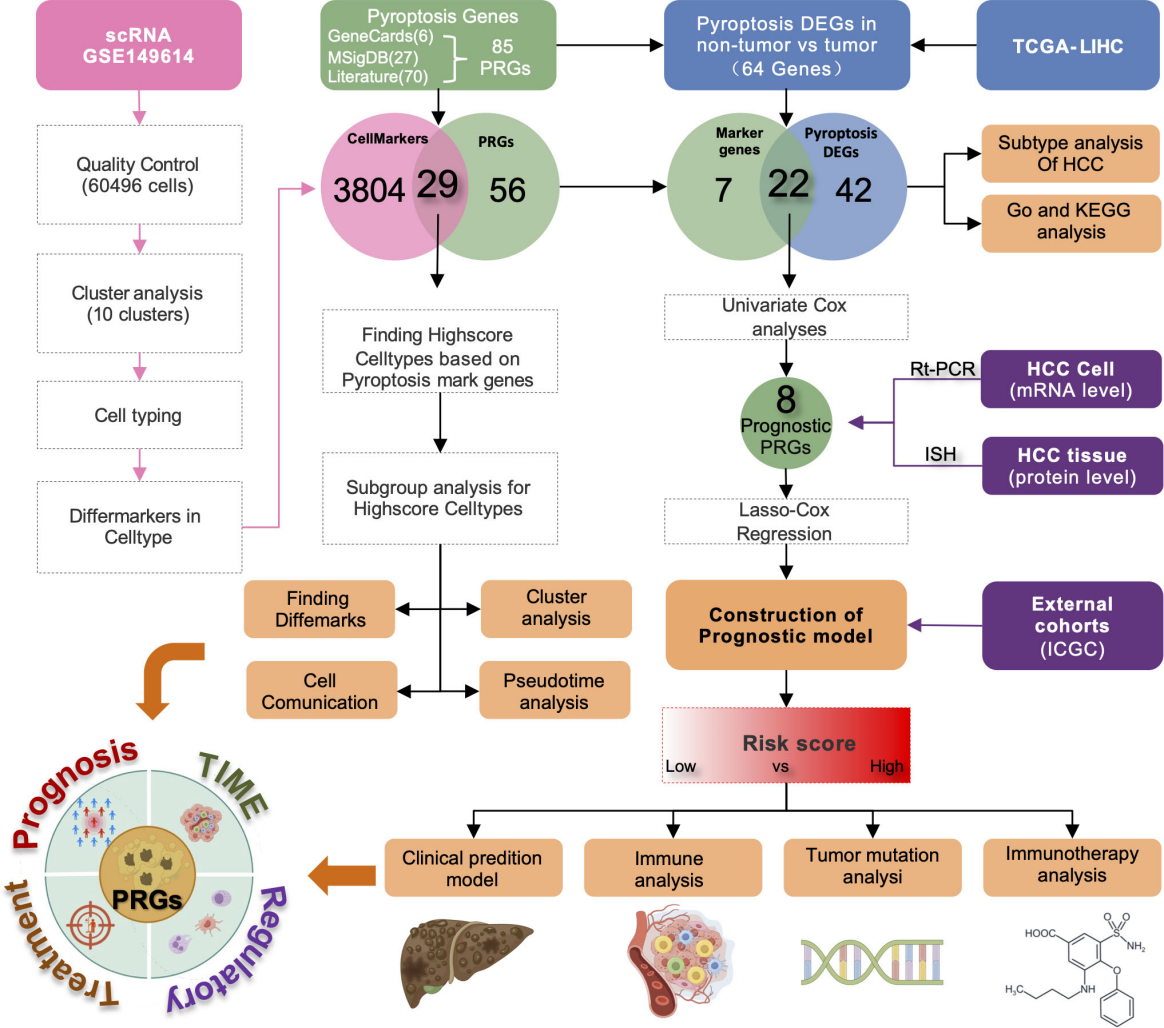
Representation of the overall flowchart of this study.

**Figure 2:**
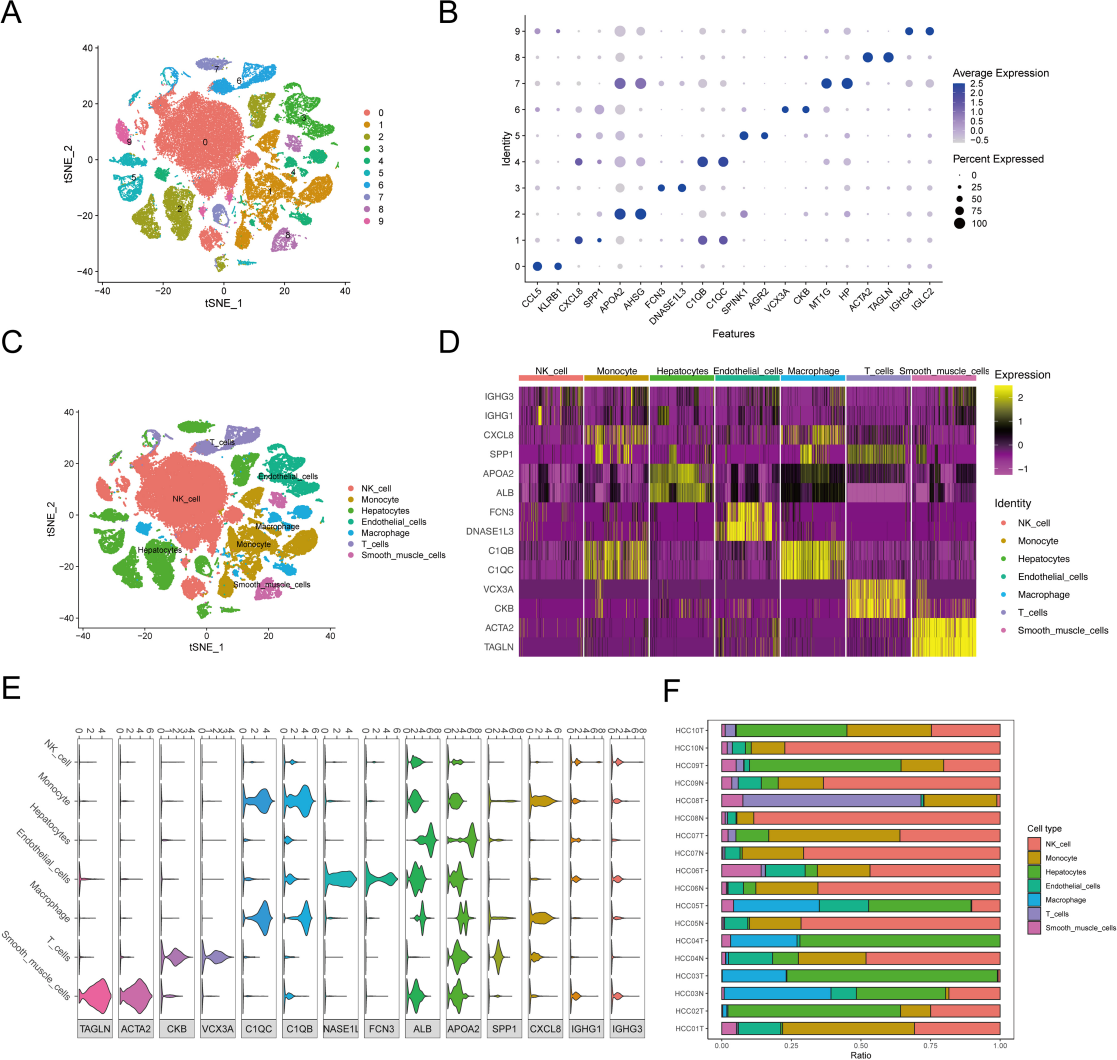
The cluster analysis and feature description of scRNA-seq data after reducing dimensions. **(A)** Cluster analysis involves reducing the dimensionality of data. Various cell clusters are represented using a stochastic distribution of t-SNE, with each cluster marked by a different color. **(B)** The expression of top2 marker genes in each cell cluster was shown by bubble map. The circle’s size indicated the gene expression proportion in the cell cluster, with darker colors corresponding to higher average expression levels. **(C)** The varied distribution of T-SNEs among various cell types. **(D)** The heat map displayed the top 2 differentially expressed genes for each type of cell. **(E)** Violin diagrams displayed the top 2 differentially expressed genes for each cell type. **(F)** The distribution of cell populations in various samples.

**Figure 1** presents the flow chart of our study. We examined scRNA-seq information from 10 primary tumor instances and eight surrounding noncancerous tissue specimens. After applying quality control criteria, we retained a total of 60,496 cells. PCA was employed to reduce dimensionality, and t-distributed stochastic neighbor embedding (t-SNE) was used to cluster the single cells; this yielded 10 optimal cell clusters (**Figure 2A**) and allowed us to identify the top two marker genes for each cluster (**Figure 2B**).

Using the SingleR package, we annotated the following seven cell types in the 10 clusters, listed from the most to least prevalent: NK cells (40.32%), hepatocytes (22.15%), monocytes (16.28%), endothelial cells (6.33%), macrophages (6.22%), T cells (5.59%), and smooth-muscle cells (3.13%) (**Figure 2C**). Violin plots and heat maps were created to display the top two DEGs across these seven cell types (**Figures 2D, E**, **Supplementary Table S5**). Additionally, we created an accumulation histogram to illustrate the proportion of each cell type across the 10 samples (**Figure 2F**).

### Expression of pyroptosis marker genes in different HCC cell types

**Figure 3:**
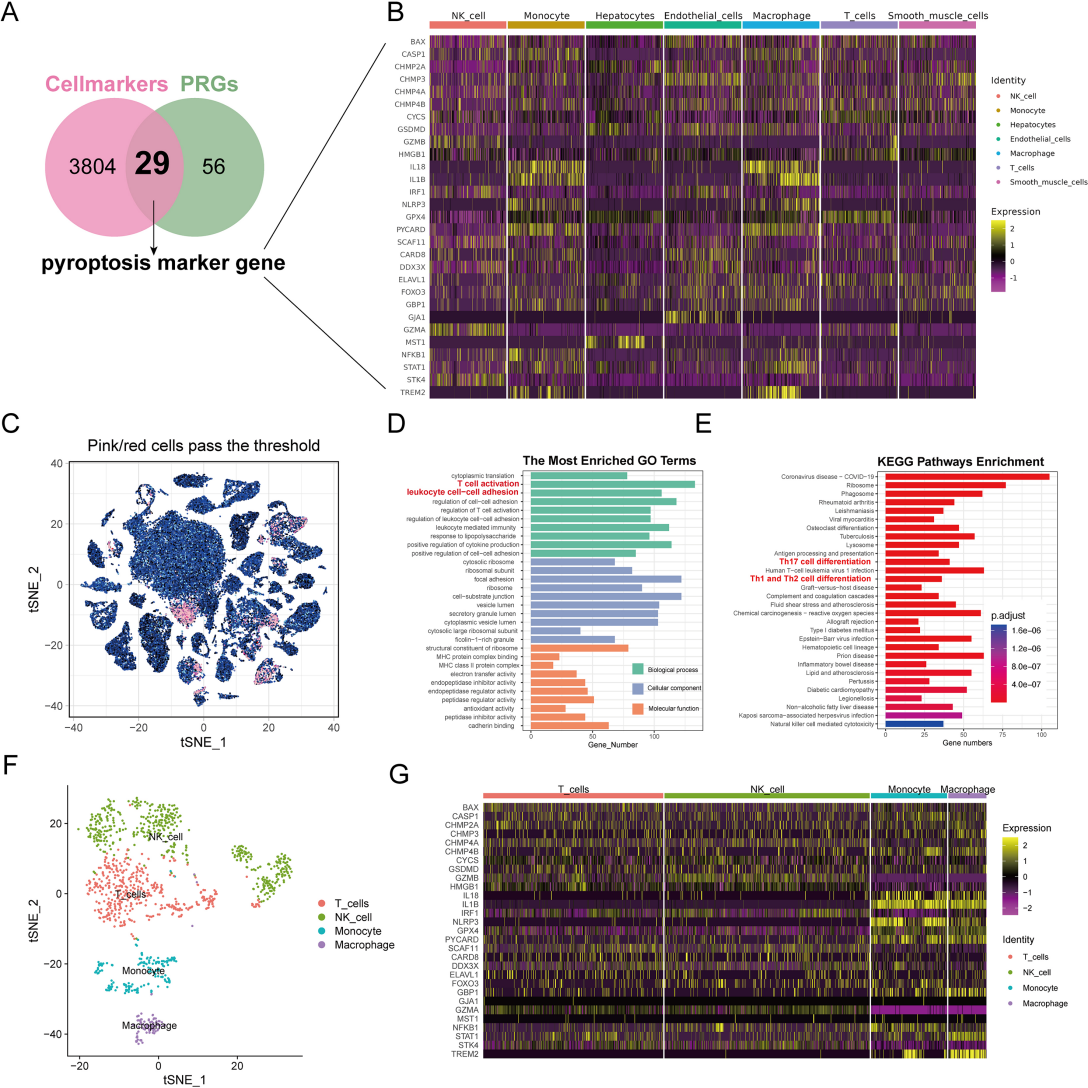
Analysis of pyroptosis marker genes for identification and enrichment of functional pathways. **(A)** The Venn diagram displays the overlap of genes associated with pyroptosis and genes that differ between cell populations. The overlapping area between the two circles is pyroptosis marker genes. **(B)** Heat maps showing the expression of pyroptosis marker genes in each cell type. **(C)** Analysis of subgroup characterized by elevated levels of pyroptosis marker genes.t-SNE visualizes the arrangement of top-ranking cells among all cells. The outcomes of GO **(D)** and KEGG **(E)** analysis for genes that are differentially expressed in highly ranked cells. **(F)** The arrangement of t-SNE for various cell types within cells with high scores. **(G)** Heat maps displayed the presence of pyroptosis indicator genes in various cell types within high-scoring cells.

Our research intersected 85 potential PRGs with DEGs across cell types, and it identified 29 key pyroptosis markers (**Figure 3A**). The heat maps displayed these genes’ expression patterns across various cell types (**Figure 3B**). The correlations among these pyroptosis markers are detailed in **Supplementary Tables S6**, **S7**, with significant positive associations noted between *STK4* and *GZMA*, *GZMB* and *GZMA*, *GSDMD* and *CHMP2A*, *GPX4* and *CHMP2A*, and *TREM2* and *IL1B*. Notably, *GZMA* was negatively correlated with *GPX4* and *CHMP2A*. All the correlations had P values below 0.05, denoting statistical significance.

### Subgroup analysis for high-score cell types based on pyroptosis marker genes

Cells with enrichment scores above 0.13 were classified as high scoring; they totaled 1,168 cells (**Figure 3C**). Subsequent clustering analysis with the SNN algorithm and cell-type identification with SingleR revealed distinct differential gene expressions (**Supplementary Table S8**). GO analysis associated the high-scoring cells with inflammatory processes, such as T-cell activation and leukocyte adhesion (**Figure 3D**, **Supplementary Table S9**). KEGG pathway analysis further indicated that genes differentially expressed in high-scoring cells were involved in inflammatory and immune-related pathways, such as Th17, Th1, and Th2 cell differentiation (**Figure 3E**). These high-scoring cells comprised mainly NK cells (41.10%), T cells (36.04%), monocytes (15.24%), and macrophages (7.62%) (**Figure 3F**). The heat maps illustrated the expression levels of pyroptosis markers in these high-scoring cells, showing elevated expression of *IL18*, *IL1B*, *NLRP3*, *GPX4*, *PYCARD*, and *TREM2* in monocytes and macrophages (**Figure 3G**).

### Cell differentiation and intercellular communication analysis of high-scoring cell types

**Figure 4:**
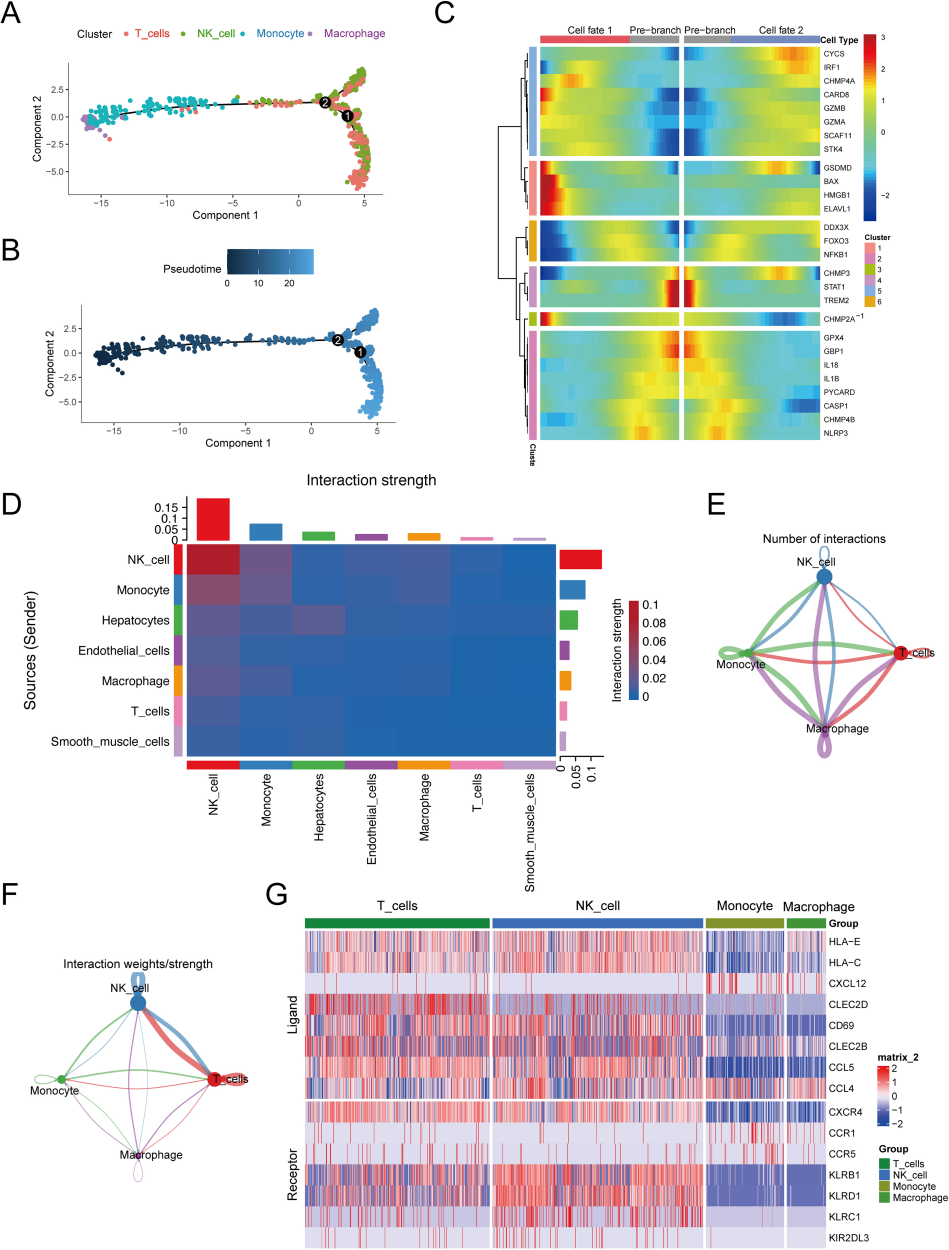
Cell differentiation and communication analysis of high-scoring cells. **(A)** Pseudo-time sequence map based on high-scoring cells. **(B)** Pseudo-time track chart, color from dark to light, indicating the time of differentiation from early to late. **(C)** Taking 2 as node, the dynamic heat map of pyroptosis differmarkers in the pseudo-sequential branch was performed. **(D)** Heat map of interaction intensity between all cell types. The redder the color, the higher the interaction intensity of the interacting ligand-receptor pairs. **(E)** Number network diagram of interaction pairs between different cell types in high-scoring cells. **(F)** The interaction between different cell types in the high-scoring cells. **(G)** Expression of parapportor receptors in different cell types in high-scoring cells.

We further conducted pseudotime trajectory analysis of the high-scoring cell types, and constructed the trajectory map of the cell types in pseudo time. The pseudo-time sequence diagram was colored from two aspects of cell types process and pseudo-time process (**Figures 4A, B**), and different branches were conducted along the direction of the pseudo-time sequence. We also used the BEAM function to analyze differences in pyroptosis marker genes between branches, visualizing them in the form of dynamic heat maps (**Figure 4C**). It is pre-branch from the second node to the root, that is, the corresponding state of macrophages and monocytes. Both Cell fate1 and Cell fate2 contain the corresponding state of T cells and NK cells. The focal extinction marker genes were clustered into 6 categories.

To delve into the communication dynamics between different cell types, our analysis first utilized heat maps to depict the interaction intensity across all cell types (see **Figure 4D**). These maps revealed the communication strength for each cell type, with the vertical axis representing the signaling senders and the horizontal axis representing the recipients. Summative bar graphs at the top and right detail the cumulative communication strength under each cell type. Notably, NK cells emerged as the most communicative, both as ligand-producing cells (weight sum = 0.133) and as receptor cells (weight sum = 0.189).

Further examination highlighted specific cell types within the high-score category, including NK cells, T cells, monocytes, and macrophages. Network diagrams were then employed to illustrate the quantity and signal intensity of interactions among these cells (**Figures 4E, F**). For instance, macrophages were observed to most frequently act upon themselves with 107 interactions, the interaction between macrophages (as receptors) and monocytes had a logarithmic interaction count of 101, and macrophages acting as ligand cells towards monocytes had a count of 100. Signal strength analysis showed that NK cells had the most potent intracellular communication (weight=0.170) and the strongest interaction with T cells when acting as receptor cells (weight=0.167).

Additionally, we found a high number of ligand-receptor pairs among these high-score cell populations, totaling 330 pairs (detailed in **Supplementary Table S10**). In a more targeted analysis, the heat map in **Figure 4G** displays the selection of the top 10 receptor-ligand pairs with the highest probability of communication, including CCL5-CCR5, CCL5-CCR1, CLEC2B-KLRB1, CLEC2C-KLRB1, CXCL12-CXCR4, HLA-E-KLRC1, HLA-E-KLRD1, HLA-C-KIR2DL3, CCL4-CCR5, and CLEC2B-KLRB1.

### Key pyroptosis genes associated with molecular subtyping of HCC

**Figure 5:**
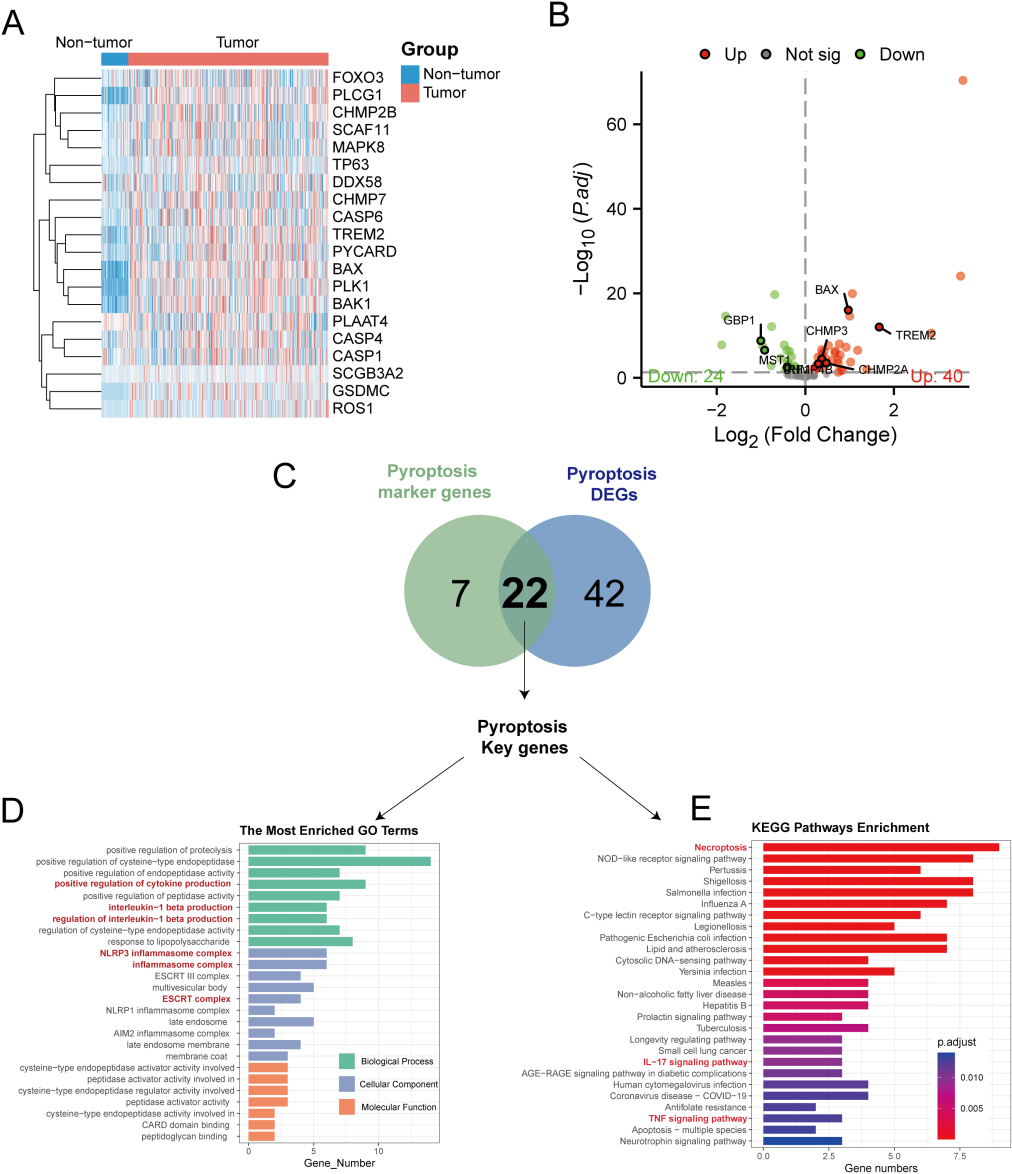
Transcriptome analysis of pyroptosis DEGs in the TCGA-LIHC dataset. **(A)** Heat maps showing the expression of pyroptosis DEGs in non-tumor vs tumor tissues. **(B)** The volcano map showed that 24 pyroptosis DEGs were significantly decreased in tumor tissues, while 40 were significantly increased. **(C)** The Venn diagram illustrates the overlap between differentially expressed genes related to pyroptosis in TCGA-LIHC and genes that serve as markers for pyroptosis in single-cell RNA sequencing, resulting in the identification of key genes involved in pyroptosis. **(D)** Conducting GO enrichment analysis on the key genes related to pyroptosis. **(E)** KEGG enrichment analysis of pyroptosis key genes.

By analyzing the TCGA-LIHC dataset and concentrating on 85 PRGs, we discovered 64 DEGs that were differentially expressed in the tumor and normal samples, with 40 genes being upregulated and 24 genes being downregulated (see **Figures 5A, B**, **Supplementary Table S11**). Cross-referencing these with the 29 pyroptosis markers yielded 22 key pyroptosis genes, including *DDX3X*, *BAX*, *IL1B*, and *TREM2* (**Figure 5C**). To clarify their functions at the molecular level, an analysis of gene ontology enrichment was performed on the genes that intersected; this revealed important biological processes mainly related to inflammation, including the promotion of cytokine production and the generation of interleukin-1 beta (**Supplementary Table S12**). The primary cellular components were linked to pyroptosis, involving entities such as the NLRP3 inflammasome complex and the ESCRT III complex (**Figure 5D**). The analysis of the KEGG pathways revealed that the identified genes were involved in signaling pathways associated with inflammation, necrosis, and apoptosis, including the TNF-signaling pathway and necroptosis (**Figure 5E**).

**Figure 6:**
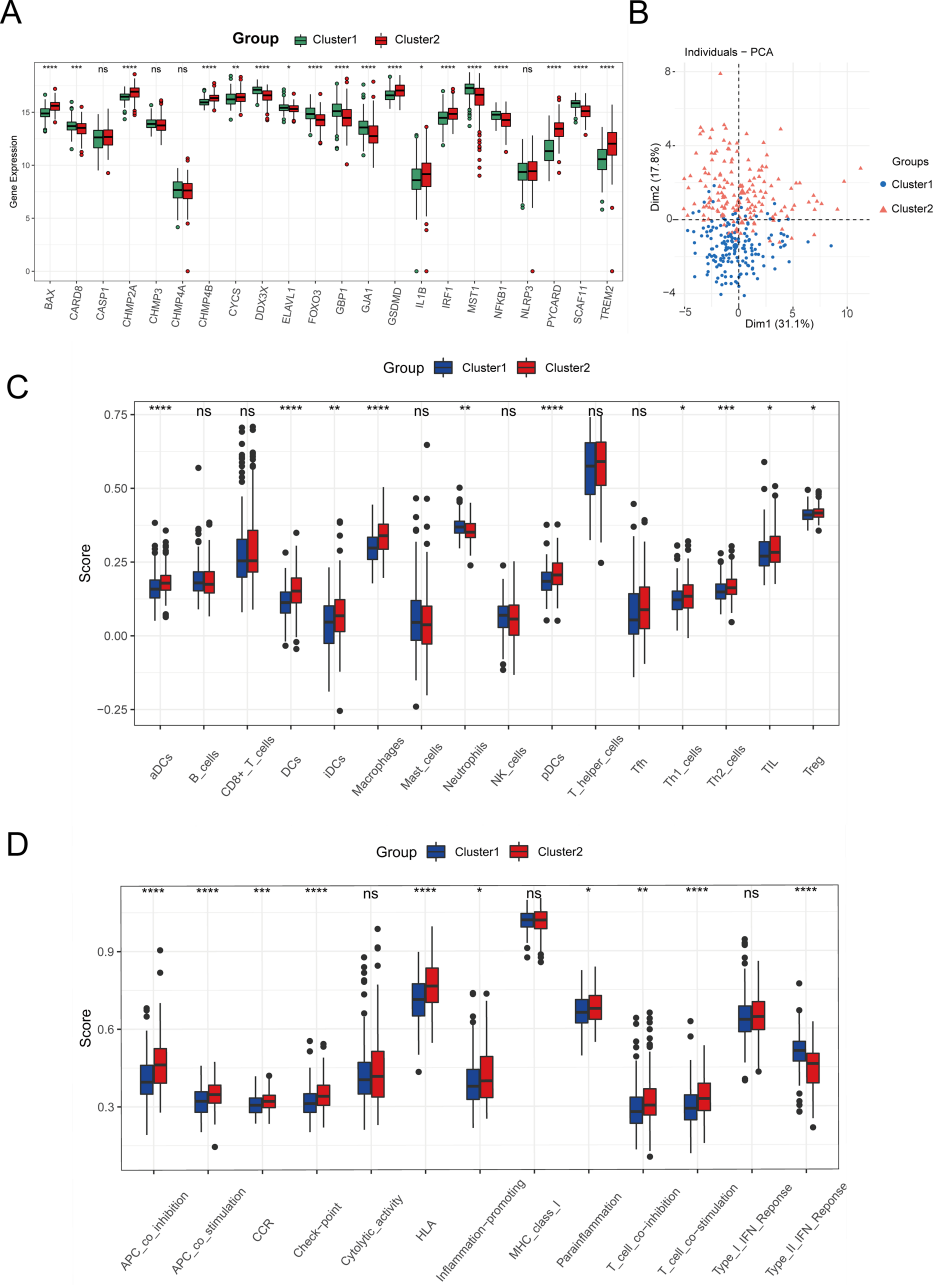
Identification of tumor subtypes. **(A)** Boxplot of the expression levels of 22 Intersection Genes in different tumor subtypes. **(B)** PCA analysis based on Intersection Genes. **(C)** The degree of infiltration of immune cells in different tumor subtypes. **(D)** Activity of immune-related pathways in different tumor subtypes. (ns: p > 0.05, *p ≤ 0.05, **p ≤ 0.01, ***p ≤ 0.001, ****p ≤ 0.0001).

To categorize TCGA-LIHC tumor samples based on 22 crucial pyroptosis genes, we employed the ConsensusClusterPlus method for consensus clustering, dividing HCC samples into two distinct subtypes (**Supplementary Figures S1A–C**). Differential expression levels of genes such as BAX, CARD8, CHMP2A, CHMP4B, and others were observed between these subtypes (**Figure 6A**). Principal component analysis (PCA) delineated that tumors from these gene intersection subtypes occupied separate regions, with Cluster 1 mainly in the third and fourth quadrants and Cluster 2 in the first and second quadrants (**Figure 6B**). Furthermore, ssGSEA analysis revealed subtype variances in the enrichment of 16 immune cells and 13 immune cell pathways. Specifically, the expressions of aDCs, DCs, iDCs, pDCs, Th1 cells, Th2 cells, TIL, and Treg were notably elevated in Cluster 2 (P < 0.05), while macrophages and neutrophils showed higher expression in Cluster 1 (P < 0.05) (**Figure 6C**). Additionally, immune pathways involving antigen presentation and inflammation were more enriched in Cluster 2 than in Cluster 1 (P < 0.05) (**Figure 6D**).

### Development and confirmation of the predictive model using the key pyroptosis genes

**Figure 7:**
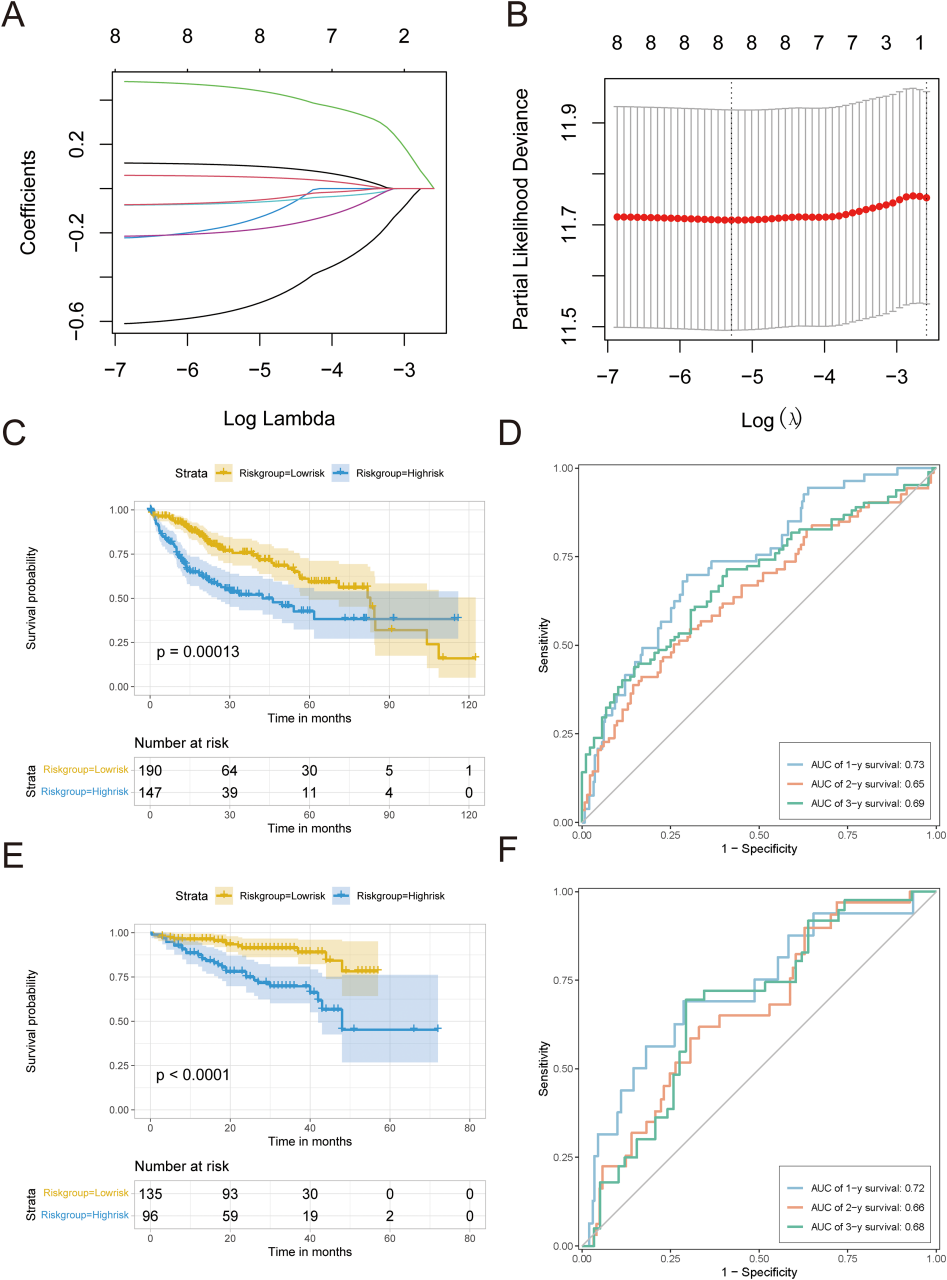
Development and confirmation of the predictive model. **(A)** LASSO analysis was used to build an appropriate model, revealing the fluctuations in lambda values of 8 prognostic genes that have a notable impact on prognosis. The X-axis represented lambda values after logization, and the Y-axis represented coefficients. **(B)** Cross-validation analysis determines the best lambda values for the fitted model. The logized lambda values are represented on the X-axis, while the model errors are represented on the Y-axis. The dotted line on the left indicates the lambda values that minimize errors and the number of features screened. **(C)** Survival curve for TCGA-LIHC. **(D)** TCGA-LIHC timeROC curve. **(E)** Survival curves of HCC data sets in ICGC. **(F)** timeROC curve of HCC data set in ICGC.A p value less than 0.05 indicated a significant difference.

We evaluated how 22 important pyroptosis genes affected the prognosis of 337 HCC patients in the TCGA-LIHC dataset. We identified eight genes (*BAX*, *TREM2*, *CHMP4B*, *CHMP3*, *GBP1*, *IRF1*, *CHMP2A*, and *MST1*) that significantly influenced HCC prognosis (P < 0.3). Utilizing LASSO-Cox regression (**Figures 7A, B**), we constructed the following risk model: risk score = (0.11836 * *BAX*) + (0.06145 * *TREM2*) + (0.49257 * *CHMP4B*) + (−0.24100 * *CHMP3*) + (−0.07721 * *GBP1*) + (−0.22166 * *IRF1*) + (−0.62887 * *CHMP2A*) + (−0.07688 * *MST1*).

The survminer package was used to establish an optimal cutoff value of 0.003, which separated patients into high-risk and low-risk groups. The Kaplan–Meier analysis showed a notable decrease in OS in the high-risk group (P < 0.05; **Figure 7C**). ROC curve analysis showed that the risk score had a strong predictive ability for one-year (AUC = 0.73), two-year (AUC = 0.65), and three-year (AUC = 0.69) OS (**Figure 7D**).

To confirm the effectiveness of the risk model, we utilized a separate group of 231 HCC patients from the ICGC database. The Kaplan–Meier analysis showed that the high-risk group had a worse OS rate (P < 0.05, as shown in **Figure 7E**), which is in line with the results from TCGA-LIHC. The time-dependent ROC assessment demonstrated comparable forecasting capability, achieving AUCs of 0.72 at one-year, 0.66 at two-year, and 0.68 at three-year OS (**Figure 7F**).

### Establishment of a prognostic nomogram for HCC

**Figure 8:**
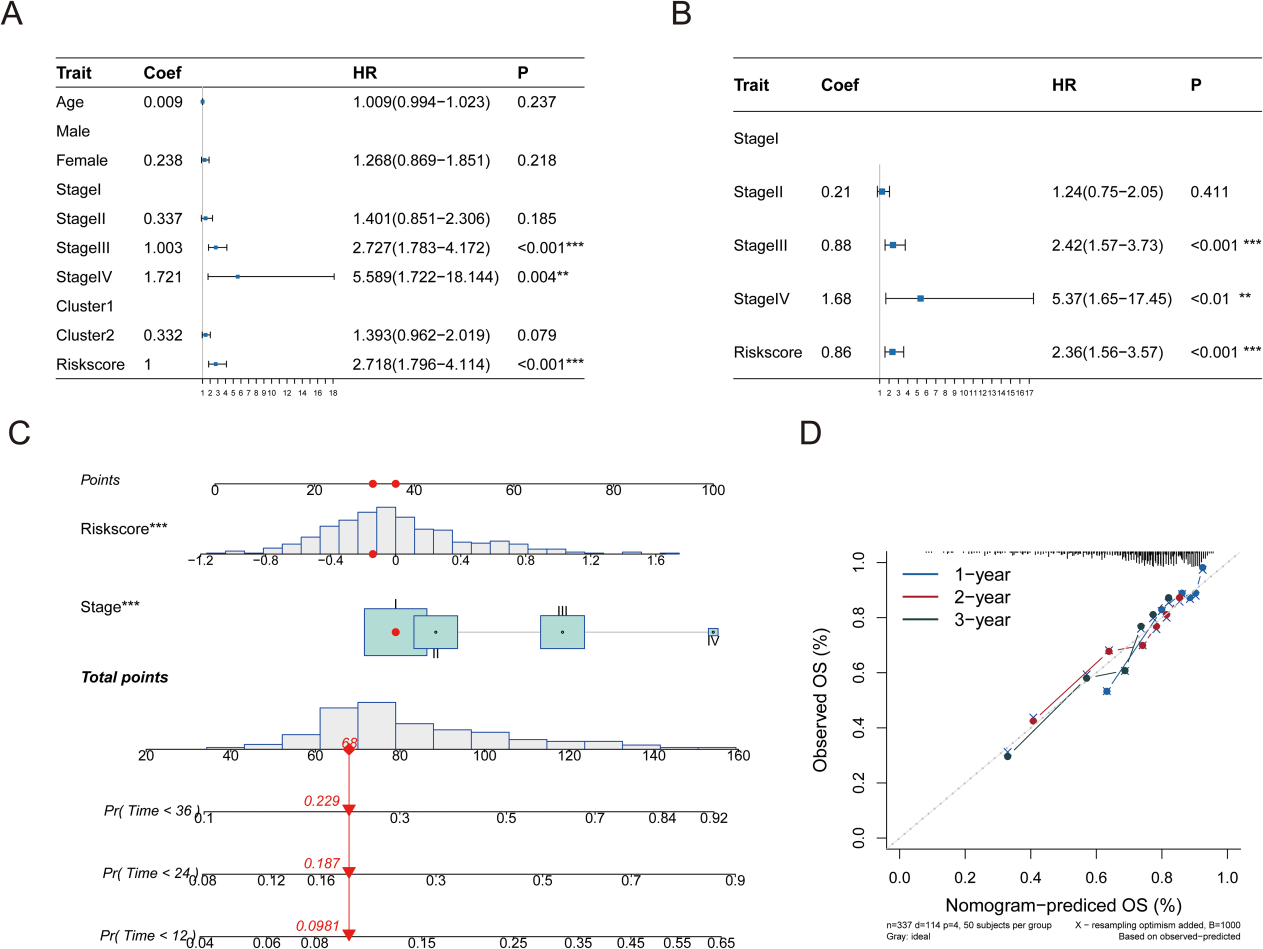
A linear model was constructed for prediction. **(A)** Performing univariate Cox regression analysis on the risk score in conjunction with hazard ratio (HR) and P-value of clinical features. **(B)** Multivariate Cox regression analysis was performed on the hazard ratio (HR) and p-value of the risk score in conjunction with clinical characteristics. **(C)** A nomogram illustrates a predictive model created using tumor stage and risk score. **(D)** Column chart’s calibration curve. The curve is close to the diagonal dotted line, indicating better prediction effect, which is an ideal column chart prediction model. Significance levels are denoted as follows: p ≤ 0.05 (*), p ≤ 0.01 (**), p ≤ 0.001 (***), p ≤ 0.0001 (****).

Next, we assessed the influence of the risk scores and various clinical characteristics on the outcomes of HCC patients. Integrating the risk scores from the pyroptosis-associated prognostic model with distinct clinical features, we initially conducted a univariate Cox analysis. The univariate forest plot indicated that the patients’ age, gender, and tumor types were proximal to the null line, which suggested their potential as risk factors for predicting HCC patient prognosis, whereas their tumor stages and pyroptosis-related risk scores were distinctly to the right of the null line, highlighting their significance (**Figure 8A**, **Supplementary Table S13**).

Following this, a multivariate Cox regression analysis using tumor stages and pyroptosis-related risk scores as independent variables revealed that both factors were situated to the right of the null line, which confirmed their status as independent risk factors for HCC prognosis (**Figure 8B**, **Supplementary Table S14**). A predictive bar chart was created to estimate the survival rate of HCC patients, which showed a high level of accuracy in distinguishing outcomes (concordance = 0.669; **Figure 8C**). Additionally, calibration curves demonstrated good concordance between the one-year, two-year, and three-year OS predictions and the patients’ actual outcomes, as depicted in the line charts of **Figure 8D**.

### Immune characteristics correlation analysis based on risk signature

**Figure 9:**
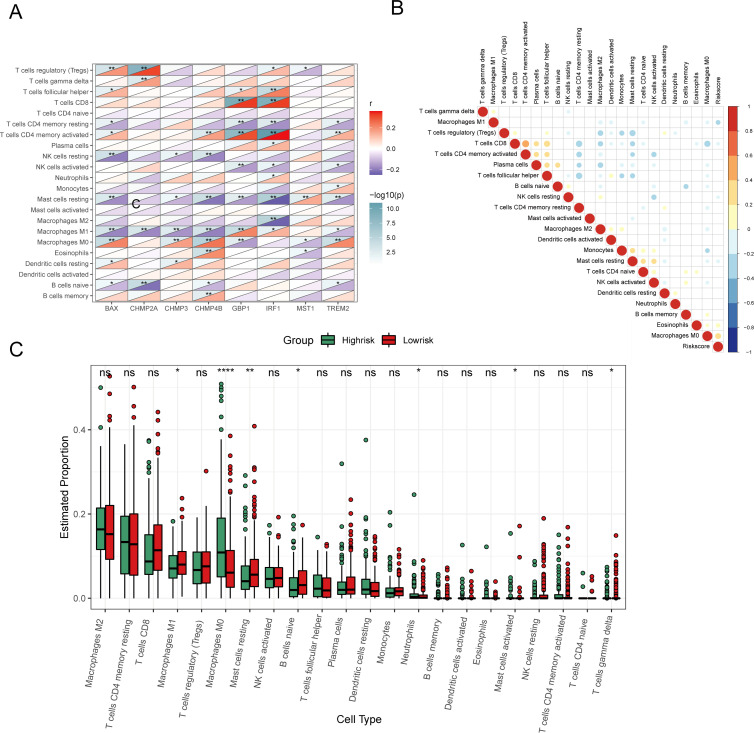
Analysis of immune characteristics of 8 prognostic genes and risk score associated with pyroptosis. **(A)** Heat map of correlation analysis between 8 prognostic genes and invasive immune cells. **(B)** Heat map of correlation analysis between risk score and invasive immune cells (positive correlation in red, negative correlation in blue, no linear correlation in white, and the larger the circle the more significant the correlation). **(C)** Analysis of different levels of immune cell infiltration between high and low risk groups. (ns: p > 0.05, *p < = 0.05 **p < = 0.01, ***p < = 0.001, ****p < = 0.0001).

To discern the relationship between key pyroptosis genes and the immune microenvironment, we employed the CIBERSORT script to assess immune cell infiltration in tissue samples using microarray expression data, determining the abundance of 22 immune cell types. Correlation analysis revealed that the eight prognostic genes from our model were closely linked with various immune cells (**Figure 9A**). Notably, CHMP4B showed a positive correlation with the abundance of memory B cells (P < 0.05), while BAX, CHMP2A, CHMP4B, and TREM2 were negatively correlated with naive B cells (P < 0.05).

Furthermore, examining the relationship between the risk score and the abundance of Macrophage M0 and Eosinophils demonstrated a positive correlation (P < 0.05), whereas a negative correlation was found with Macrophage M1 (P < 0.05) (**Figure 9B**). Other immune cells did not show a significant correlation. Boxplots comparing immune cell proportions between high- and low-risk groups highlighted that low-risk samples had significantly higher scores for Macrophages M1, resting Mast Cells, naive B cells, and other cell types. Conversely, high-risk samples had elevated scores for Macrophages M0, Neutrophils, and activated Mast cells (P < 0.05, **Figure 9C**).

### Tumor mutation analysis based on risk signature

**Figure 10:**
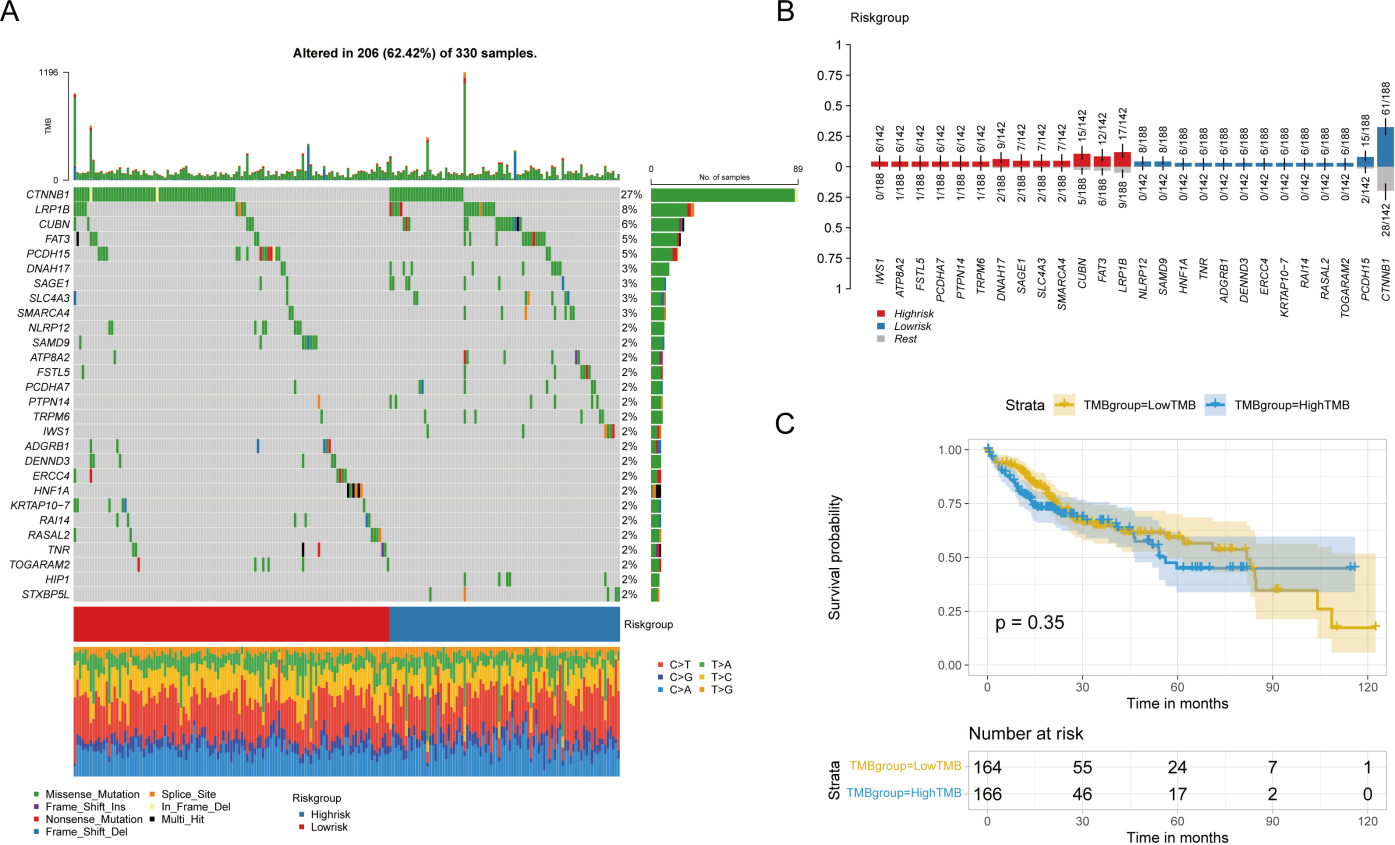
Influence of risk groups on genetic variation in LIHC patients **(A)** Waterfall diagram shows genes with significant mutation differences between high and low risk groups. **(B)** The bar chart shows the degree of enrichment of genes with distinct mutation differences in the high-low risk group. **(C)** Survival differences between patients with high and low TMB.

We analyzed the effect of pyroptosis-related risk scores on genetic variants in HCC patients, examining somatic mutation data. Key genes, such as CTNNB1, LRP1B, CUBN, and FAT3, exhibited significant mutational differences between high- and low-risk groups (P < 0.05) (**Figure 10A**). A boxplot showed the prevalence of gene mutations, with IWS1, ATP8B2 (corrected from ATPBA2), and FSTL5 mutations occurring more frequently in high-risk patients, and CTNNB1, PCDH15, and TOGARAM2 more common in low-risk patients (**Figure 10B**).

Tumor mutation burden (TMB) was calculated for each patient, and patients were categorized into high or low TMB groups based on the median TMB; however, no significant differences in overall survival (OS) were observed (**Figure 10C**). Similarly, no statistical differences in TMB or MSI were found between high and low-risk groups, nor was there a linear correlation with risk scores (**Supplementary Figures S2A–D**).

**Figure 11:**
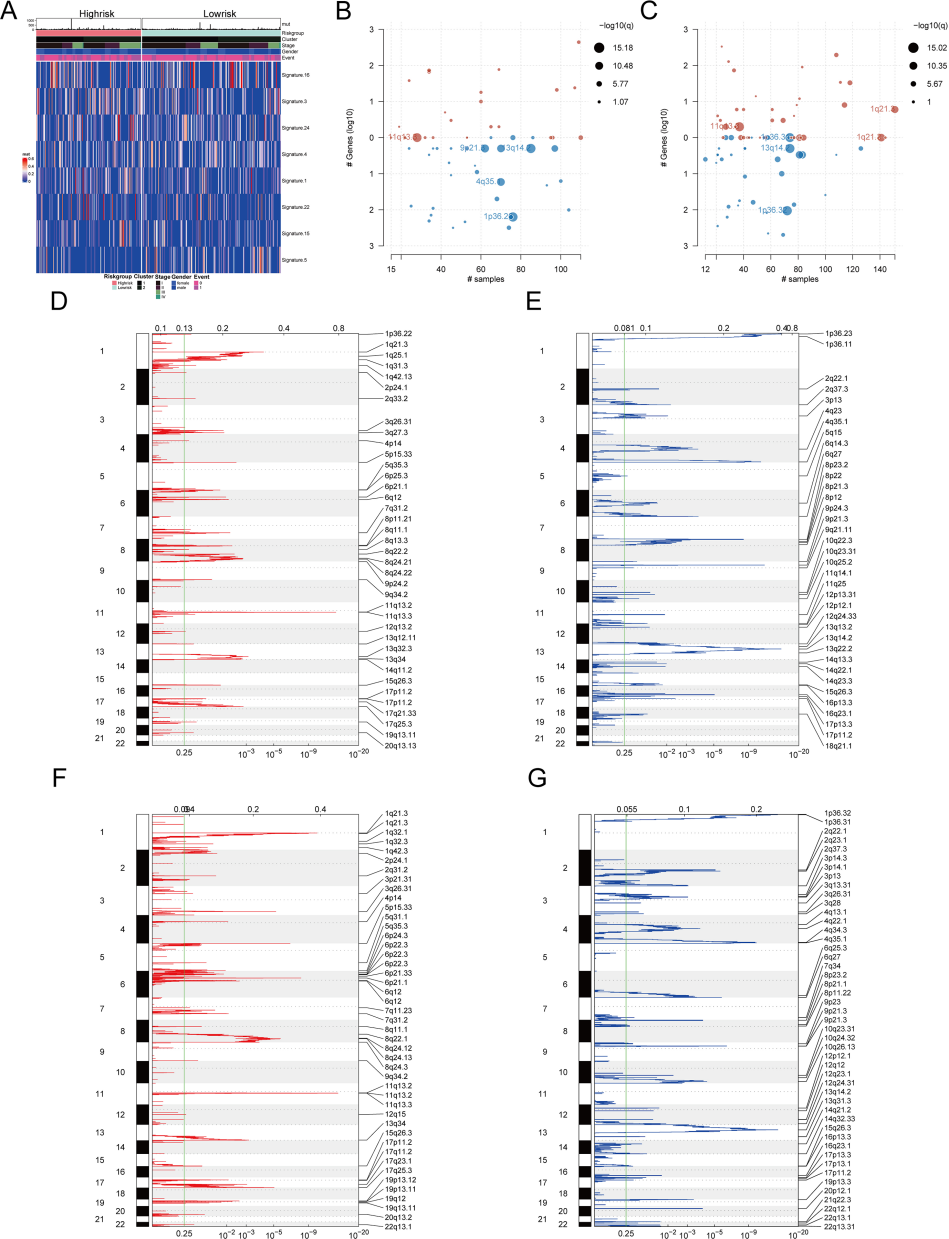
Mutation characteristics and copy number variation analysis of different risk groups **(A)** Heat map showed significant mutation characteristics between high and low risk groups (top8). **(B)** Gene segments with significant copy number variation in the high-risk group. **(C)** Gene segments with significant copy number variation at low risk. **(D)** Gene segments with increased copy numbers at high risk. **(E)** Gene segments with copy number deletion at high risk. **(F)** Gene segments with increased copy numbers at low risk. **(G)** Gene segments with copy number deletion at low risk.

Furthermore, mutation signatures 16, 3, 24, 4, 1, 22, 15, and 5 were prevalent in the tumor samples (**Figure 11A**). Using GISTIC 2.0 for copy number variation data analysis and maftools for visualization, we noted that high-risk patients had significant gene copy number deletions at 1p32.3, 4q35.1, 9p21.3, and 13q14.2, with amplifications at 11q13.3 (**Figure 11B**). In low-risk patients, significant deletions were at 1p32.3, 1p32.2, and 13q14.2, and amplifications were at 1q21.3 and 11q13.3 (**Figure 11C**). Additionally, high-risk patients had significantly more CNV alterations than low-risk patients (**Figures 11D–G**).

### Drug sensitivity of HCC patients based on pyroptosis-related prognostic genes

**Figure 12:**
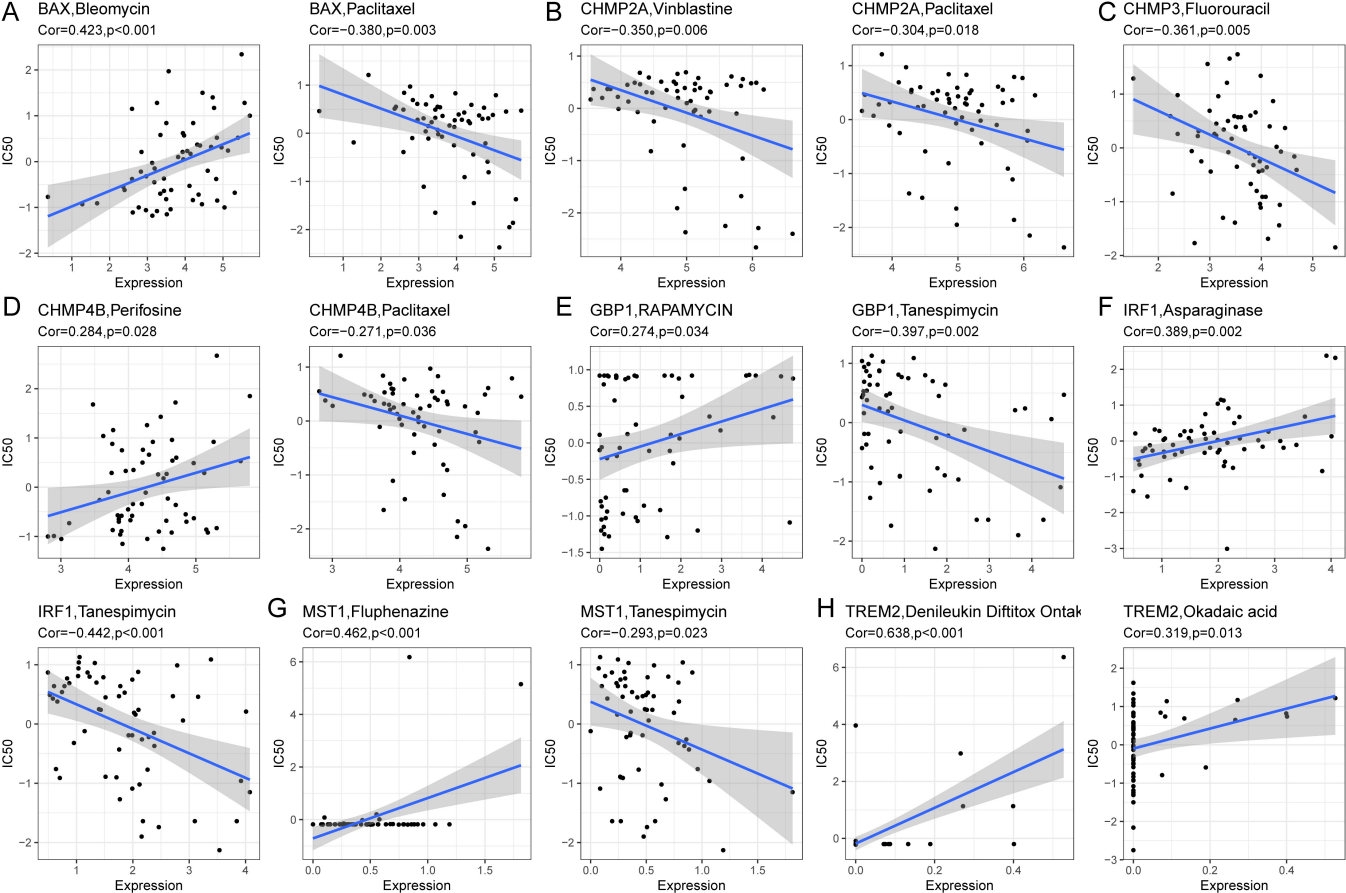
Correlation analysis between prognostic genes and IC50 of drugs. **(A)** BAX was positively correlated with IC50 of Bleomycin and negatively correlated with IC50 of Paclitaxel. **(B)** CHMP2A was negatively correlated with IC50 of Vinblastine and Paclitaxel. **(C)** CHMP3 was negatively correlated with IC50 for Fluorouracil. **(D)** CHMP4B was positively correlated with IC50 of Perifosine and negatively correlated with IC50 of Paclitaxel. **(E)** GBP1 was positively correlated with IC50 of Rapamycin and negatively correlated with IC50 of Tanespimycin. **(F)** IRF1 was positively correlated with IC50 of Asparaginase and negatively correlated with IC50 of Tanespimycin. **(G)** MST1 was positively correlated with IC50 of Fluphenazine and negatively correlated with IC50 of Tanespimycin. **(H)** TREM2 was positively associated with IC50 of DenileukinDiftitoxOntak and Okadaic acid.

To determine if pyroptosis-related prognostic genes could assess HCC treatment sensitivity, we utilized gene expression data from 60 cell lines and IC50 values for 24,360 drugs from the CellMiner database, excluding any with incomplete information. We correlated the expression of eight prognostic pyroptosis genes with the IC50 values of 62 drugs. A significant correlation was found between the IC50 values of 45 drugs and the gene expressions (P < 0.05, **Supplementary Table S15**). BAX showed a positive correlation with the IC50 values of seven drugs, including Bleomycin (R = 0.423) and Floxuridine (R = 0.362), and a negative correlation with Paclitaxel (R = -0.380) and Eribulin mesylate (R = -0.295). CHMP2A exhibited a negative correlation with Vinblastine (R = -0.350) and Paclitaxel (R = -0.304). CHMP3’s expression was inversely related to the IC50 for Fluorouracil (R = -0.361). CHMP4B was positively correlated with Perifosine (R = 0.284) but negatively with Paclitaxel (R = -0.271) and Tanespimycin (R = -0.256). TREM2’s expression positively matched the IC50 of drugs such as Denileukin Diftitox Ontak (R = 0.638) and Okadaic acid (R = 0.320). These gene-drug correlations were visually represented (**Figures 12A–H**), with all p-values below 0.05.

### Evaluating the immunotherapy based on the risk signatures

**Figure 13:**
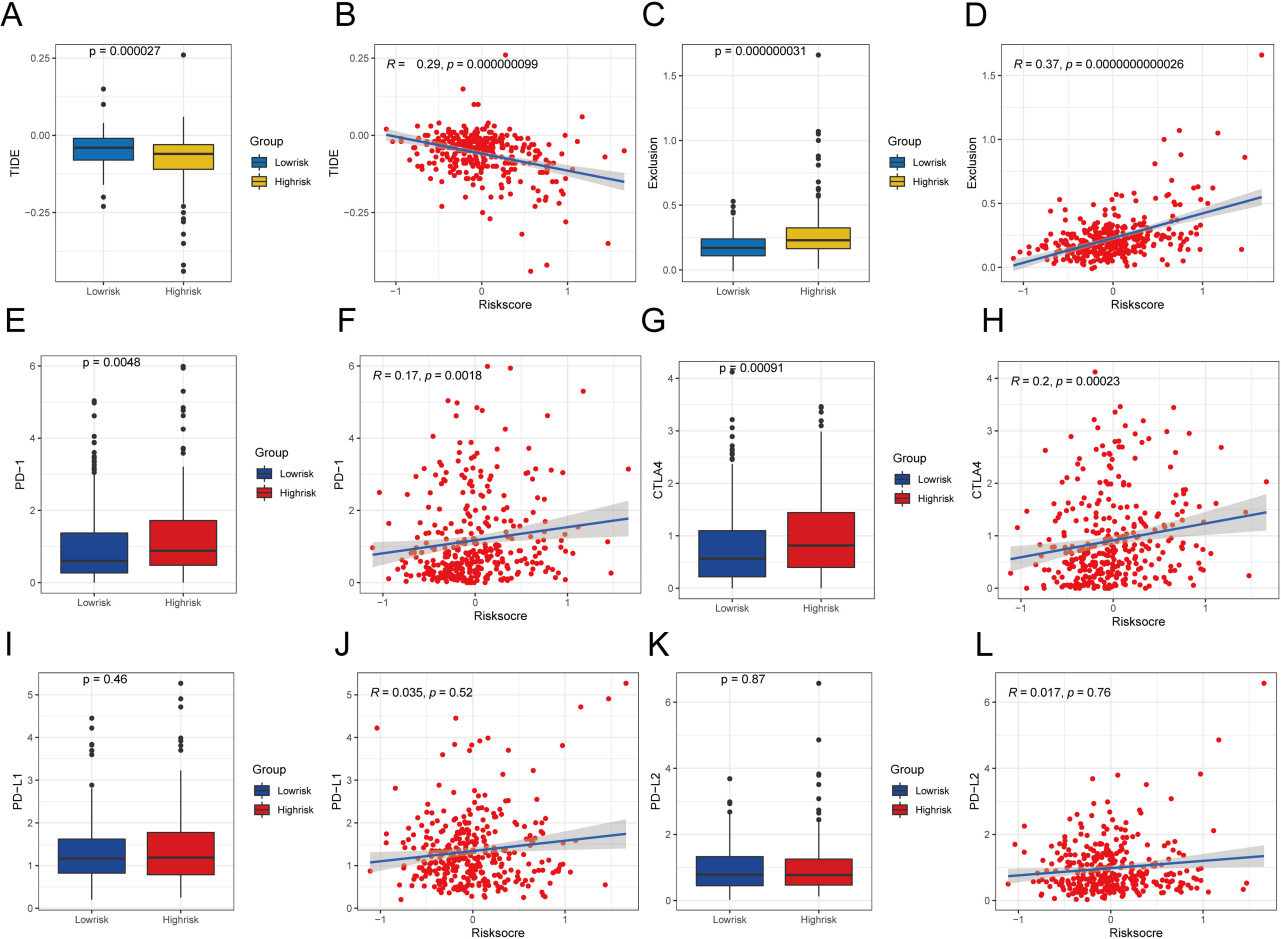
Comparative analysis of TIDE scores and immune checkpoint expression in various risk categories. **(A)** Difference of TIDE among different risk groups. **(B)** Examining the relationship between TIDE and the level of risk. **(C)** Exclusion differences among different risk groups. **(D)** Examining the relationship between exclusion score and the level of risk **(E)**Contrasts in PD-1 levels among groups with high and low risks. **(F)** Correlation analysis between PD-1 expression and risk score. **(G)** Differences in CTLA4 expression between different risk groups. **(H)** Correlation analysis between CTLA4 expression and risk score. **(I)** Variations in PD-L1 expression across various risk categories. **(G)** Correlation analysis between PD-L1 expression and risk score. **(K)** Expression difference of PD-L2 between different risk groups. **(L)** Correlation analysis between PD-L2 expression and risk score. A significance level of less than 0.05 was deemed as indicating a difference.

First, we determined the immunotherapy sensitivity for the high- and low-risk patient groups using the TIDE algorithm. The group at high risk had lower TIDE scores than the group at low risk (P < 0.05; **Figure 13A**), and it had a negative correlation with the risk score (R = −0.29, P < 0.05; **Figure 13B**), which suggests an improved response to immunotherapy. Moreover, the immune evasion strength, as indicated by the exclusion score, was elevated in the group at high risk (P < 0.05; **Figure 13C**), and it exhibited a positive association with the risk score (R = 0.37, P < 0.05; **Figure 13D**).

To evaluate the sensitivity of the immunotherapy, we analyzed the relationships between typical immune checkpoints (PD-1, PD-L1, PD-L2, and CTLA4) and the risk scores. In the high-risk group, there was a notable increase in the expression of PD-1 and CTLA4, which were also found to be positively correlated with the risk score (PD-1 R = 0.17, CTLA4 R = 0.20). This can be seen in **Figures 13E, F, G, H**, with a significance level of P < 0.05. Nevertheless, there were no notable variations in the expression of PD-L1 and PD-L2 between the two risk groups (P > 0.05; **Figures 13I, K**), and these immune checkpoints did not show direct relationships with the risk scores (P > 0.05; **Figures 13J**, **L**).

### Validating the expression of PRGs in HCC cells and tissue samples

**Figure 14:**
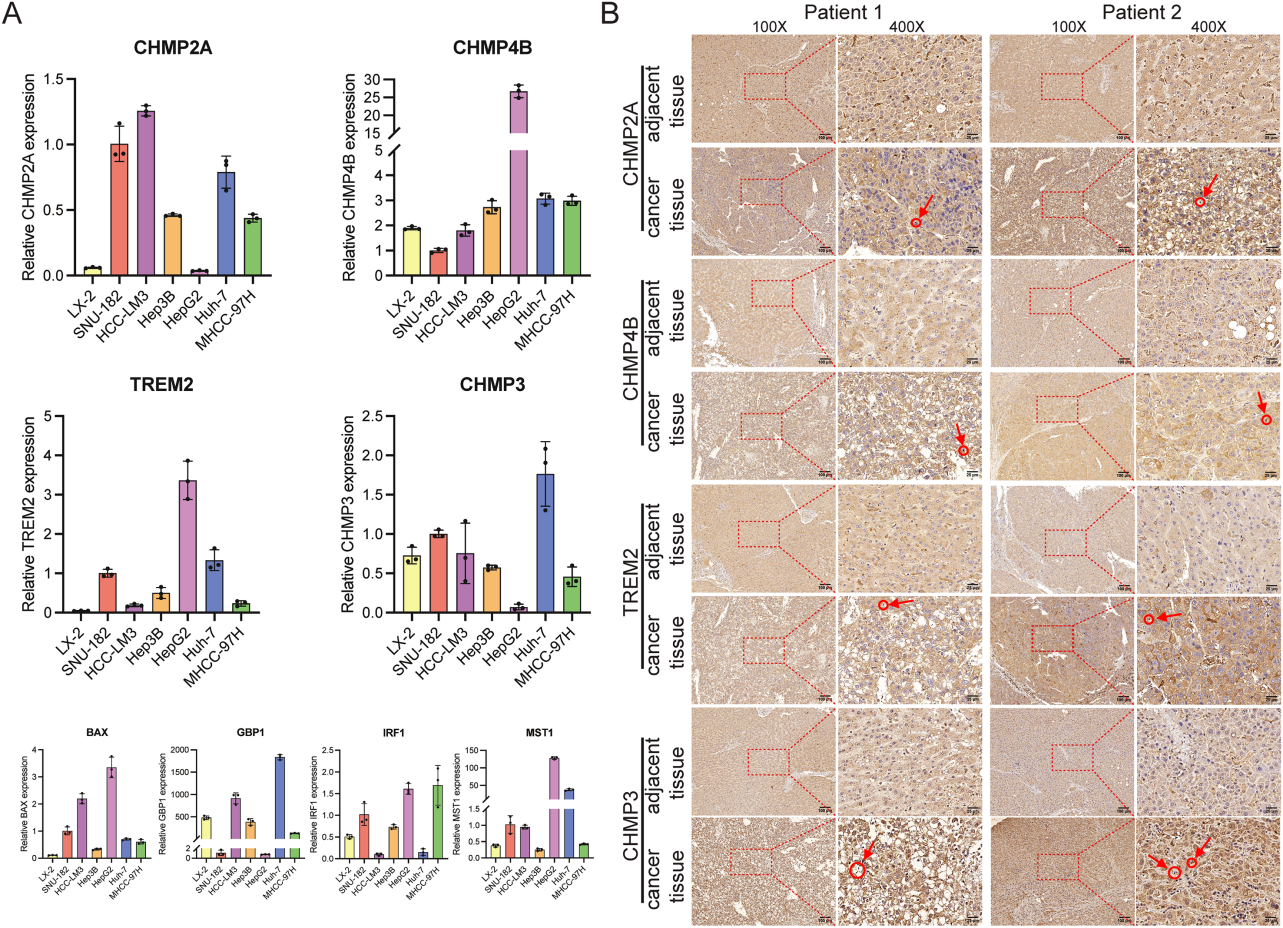
The manifestation of predictive genes in hepatocellular carcinoma cells and tissues. **(A)** *In vitro*, PRGs mRNA levels were measured using RT-qPCR. Expression of the eight prognostic PRGs was measured by qRT-PCR. The LX-2 cell line was used as a normal-like liver cell control, while SUN-182, HCC-LM3, Hep3B, HepG2, Huh-7, and MHCC-97H are established HCC cell lines. **(B)** IHC staining demonstrated significantly higher expression of CHMP2A, CHMP4B, TREM2, and CHMP3 in HCC tumor tissues compared to adjacent non-tumor tissues. The circled regions, indicated by arrows, highlight the positive immune cells.

We conducted qRT-PCR analysis to investigate the expression levels of *BAX*, *CHMP4B*, *TREM2*, *CHMP3*, *GBP1*, *IRF1*, *CHMP2A*, and *MST1* in HCC cells (primer sequences: **Supplementary Table S16**). The results indicate a notable increase in the expression of these genes in HCC cells when compared to normal liver cells, as shown in **Figure 14A**. After finding increased PRG expression in the HCC cell lines, we analyzed the levels of *CHMP2A*, *CHMP4B*, *TREM2*, and *CHMP3* in the HCC and nearby normal tissue samples of two patients through immunohistochemistry (IHC). The IHC analysis showed pronounced overexpression of *CHMP2A*, *CHMP4B*, *TREM2*, and *CHMP3* in the HCC tissue samples, which is consistent with the qRT-PCR results. Notably, positive staining was also observed in smaller, scattered infiltrating cells morphologically consistent with immune cells, which aligns with our scRNA-seq findings. (**Figure 14B**).

## Discussion

In HCC, the dysregulation of PRGs has been implicated in tumor progression and patient prognosis, suggesting that these genes could serve as biomarkers or therapeutic targets (14–16). Leveraging scRNA-seq technology, this study characterized PRG expression heterogeneity across immune cell populations in HCC tumor and adjacent tissues. We observed significant PRG enrichment in NK cells, monocytes, macrophages, and T cells, with expression patterns varying among cellular subtypes and correlating with immune infiltration levels. These findings reveal the cellular heterogeneity of PRG expression and its association with immune microenvironment remodeling, providing a foundation for exploring PRGs’ functional roles and clinical significance in HCC. Furthermore, our prognostic model offers a novel approach for HCC risk stratification, though the underlying mechanisms require experimental validation.

Single-cell analysis demonstrated predominant enrichment of PRGs within immune cell populations—notably NK cells, monocytes, macrophages, and T cells. Functional enrichment confirmed these genes primarily regulate inflammatory and immune signaling pathways. Intercellular communication analysis further revealed extensive crosstalk among PRG-enriched immune subsets. Collectively, these findings suggest that pyroptosis may serve as a critical nexus between HCC’s immune landscape and tumor microenvironment communication. Leveraging these key genes, we stratified TCGA HCC samples into two distinct molecular subtypes exhibiting divergent immune infiltration patterns, directly linking pyroptosis to tumor heterogeneity. This work comprehensively maps the cellular heterogeneity of pyroptosis-related genes in HCC and defines their association with immune subtypes, providing a framework for investigating pyroptosis in tumor immunity.

The heterogeneity of the tumor ecosystem complicates the prediction of HCC prognosis and the effectiveness of treatments (38). In this study, we found eight prognostic PRGs, including *TREM2*, *CHMP4B*, *CHMP3*, and *CHMP2A*; we also confirmed their high expression levels in HCC cells and tissue samples. Recently, scholars have highlighted the role of myeloid cells, with *TREM2* emerging as a key immune signaling hub (39). High *TREM2* expression is associated with poorer outcomes in various cancers, including HCC (40–42). In one study, HCC patients with TREM2+ macrophages had significantly shorter survival, suggesting that *TREM2* has immunosuppressive activity and promotes immune escape in HCC (43). Our findings point to the upregulation of *TREM2* in HCC, which indicates its potential as a therapeutic target to counteract immunosuppression. Furthermore, *CHMP4B*, *CHMP3*, and *CHMP2A*, which are members of the CHMP protein family, contribute to HCC development. Similarly, *CHMP3* and *CHMP2A* show increased expression in HCC tissue samples, thus potentially influencing tumor progression (44, 45). These findings underscore the significance of these genes as potential therapeutic targets in HCC treatment.

Given the poor prognosis for most HCC patients, along with the risks of metastasis and recurrence, establishing an effective predictive model is crucial for evaluating patient outcomes. Researchers have previously constructed various prognostic risk models for HCC based on different gene functions, such as those related to TP53 (46), ferroptosis (47), anoikis (48), MVI-related genes (49), and earlier pyroptosis-related gene models (23, 50, 51). This study developed a risk score model based on eight prognosis-related pyroptosis genes and stratified patients into high- and low-risk groups based on this score. Further analysis showed that the low-risk group had a significantly better prognosis than the high-risk group. Compared to the aforementioned models, the model in this study demonstrated stable and superior predictive ability in both the TCGA-LIHC and the external ICGC validation cohorts, with 1-, 3-, and 5-year AUC values of 0.73, 0.65, and 0.69, respectively, outperforming some previously reported models based on single molecules or specific pathways (23, 46–51). Furthermore, this study not only confirmed the independent prognostic value of the model through multivariate analysis but also deepened the mechanistic understanding of HCC development by integrating features of the immune microenvironment at the single-cell level. The constructed nomogram, which incorporates staging and the risk score, showed good discrimination and calibration, further enhancing the model’s clinical applicability. Therefore, the PRG risk model established in this study holds advantages in both biological mechanism analysis and clinical translation, providing a new tool for HCC prognosis evaluation and personalized treatment decisions.

While the AUCs of the eight gene signature are moderate, the integration of our risk score with tumor stage in the final nomogram enhances its predictive accuracy. Future iterations could further improve this by incorporating additional clinical variables, such as serum AFP levels or specific mutation statuses, to create an even more powerful tool for clinical decision-making. It is also important to note that while our 8-gene risk signature was validated externally, the combined nomogram itself was not, due to a lack of harmonized clinical staging data in the validation cohort. Therefore, the nomogram requires further validation in independent or prospective cohorts with complete clinical information.

Tumor-infiltrating immune cells (TICs) regulate the function of cancer cells in the TME (52), potentially laying the groundwork for immunotherapy. HCC exhibits a complex TME that contributes to tumor heterogeneity and malignant properties. Immune suppression in the liver may promote immune escape in HCC (53, 54). Our preliminary results show a strong correlation between three CHMP protein family genes and the infiltration levels of multiple immune cell types in HCC samples, with memory B cell abundance positively correlating with CHMP4B, whereas naive B cell abundance negatively correlates with BAX, CHMP2A, CHMP4B, and TREM2. To further understand the implications of our risk signature, we analyzed the correlation between the risk score and the abundance of Macrophage M0 and Eosinophils. The risk score positively correlates with Macrophage M0 and Eosinophils but negatively correlates with Macrophage M1. In low-risk samples, Macrophages M1, resting Mast Cells, and naive B cells exhibit significantly higher immune infiltration scores, while high-risk samples show significantly higher scores for Macrophages M0, Neutrophils, and activated Mast cells. Tumor-associated macrophages are a critical component of the TME (55). Additionally, a recent study has revealed that the density of resting Mast cells is significantly higher in low-risk groups than in high-risk groups, according to their prognostic signature in HCC (56). These findings suggest that our risk signature, based on PRGs, could independently predict the immune infiltration state of HCC and may become the basis for the development of immunotherapy for HCC.

For personalized cancer treatment, precise identification of genomic alterations is crucial. This study evaluated the impact of pyroptosis-related risk scores on genetic variant levels, including SNPs and CNVs. We discovered that mutations in IWS1, ATP8B2, and FSTL5 predominantly occurred in high-risk patients, whereas mutations in CTNNB1, PCDH15, and TOGARAM2 were mainly found in low-risk patients. According to publicly available data, IWS1 is ubiquitous across mammalian species (www.proteinatlas.org), and IWS1 phosphorylation significantly alters tumor cell biology (57). CTNNB1 mutations have been associated with multiple cancers, and in HCC, these mutations were significantly linked to a better prognosis (58), aligning with our findings. We also investigated the association between risk scores and TMB, MSI, or CNV, finding no statistical differences in TMB and MSI between the high and low-risk groups. However, the high-risk group exhibited significantly more CNV alterations than the low-risk group. Thus, our risk model could facilitate the identification of specific gene mutations to provide tailored treatment approaches.

Our immunotherapy assessment revealed distinct patterns in high-risk HCC patients. This group demonstrated lower TIDE scores that negatively correlated with risk scores, suggesting greater baseline potential for treatment response. However, these patients simultaneously exhibited elevated T cell exclusion scores showing positive correlation with risk scores, indicating significant immune evasion. This apparent contradiction between response potential and escape mechanisms reflects complex tumor microenvironment dynamics requiring further investigation. Recently, immune checkpoint inhibitors (ICIs) based on programmed cell death protein 1 (PD-1)/programmed cell death ligand 1 (PD-L1), combined with targeted drugs and local therapies, have advanced the systemic treatment of advanced HCC (59, 60). Our study found that the high-risk patients showed significantly increased PD-1 and CTLA4 expression with positive correlations to risk scores, aligning with pyroptosis-mediated immunogenicity enhancement (61). In contrast, PD-L1 and PD-L2 expression remained unchanged across risk groups and showed no score correlation. These patterns specifically justify exploring PD-1/CTLA4 inhibitors for high-risk HCC, particularly when combined with evasion-countering approaches.While the TIDE results and checkpoint profiles suggest treatment potential, TIDE’s predictive framework originated from melanoma and NSCLC studies with limited HCC validation. The observed contradiction between low TIDE scores and high exclusion scores in our high-risk group further underscores HCC’s unique immunotherapy prediction challenges. Thus, while biologically plausible, our TIDE-derived predictions remain hypothesis-generating and require validation through prospective HCC immunotherapy trials with documented outcomes.

Future validation of the prognostic model may leverage higher-resolution data. Cross-validation against single-cell resources—such as the DRMref drug resistance atlas (62)—could assess whether the 8-gene signature consistently marks resistance-associated states across cancer contexts. Spatial transcriptomics analysis (e.g., SpaRx-based methods applied to risk-stratified tumors (63)) may further map signature gene co-localization within resistant niches and characterize distinct spatial ecosystems in high-risk tumors. Such approaches would connect bulk-tissue prognostication with spatial heterogeneity in drug response.

This study has several strengths, including the novel use of scRNA-seq data to inform a prognostic model and the validation of key gene expression patterns. However, we also recognize certain limitations. First, functional validation is crucial for elucidating the molecular mechanisms of the identified PRGs. Our qRT-PCR and IHC experiments provided initial biological validation, confirming dysregulation of key model genes (e.g., CHMP2A, CHMP4B, TREM2) in HCC cells and tissues. We acknowledge these assays are descriptive and their scope was limited by sample availability. To address this and extend our findings, future functional studies will include conducting gene knockout (e.g., via CRISPR-Cas9) and overexpression experiments in HCC cell lines, performing mechanistic assays (such as LDH release and caspase-1 activation) to assess impact on pyroptosis induction, and investigating effects on immune cell recruitment and function using T-cell co-culture systems. This staged approach—first establishing a robust prognostic signature from high-throughput omics data, followed by targeted functional validation—represents a well-established workflow in the field, and will be the focus of our future work. Second, this study did not characterize the spatial architecture of PRG-high immune cells in relation to tumor nests. Spatial transcriptomics or multiplex immunohistochemistry (mIHC) would be necessary to delineate these critical cellular interactions within the tumor microenvironment. This spatial dimension represents an important aspect for understanding immune evasion mechanisms and therapeutic resistance. Third, the established risk model requires validation in larger, multi-center prospective clinical trials to confirm its clinical utility. Its potential for predicting immune checkpoint inhibitor efficacy could not be assessed due to the lack of relevant immunotherapy cohort data and warrants future investigation. Translating these findings into clinical practice will be a focus of future work. The biological plausibility of the model is supported by the well-established roles of many included PRGs (e.g., in inflammasome complexes and cytokine production) in pyroptosis and immune signaling.

## Conclusions

This study established a prognostic model based on PRGs, thus offering valuable insights into the immune microenvironment of HCC. The findings underscore the potential of this model to inform personalized treatment strategies and improve prognostic assessments, thereby enhancing patient management. Future research should focus on validating our results by including larger cohorts and additional clinical parameters, thus ultimately facilitating the translation of these results into clinical practice for better patient outcomes.

## Data Availability

The original contributions presented in the study are included in the article/**Supplementary Material**. Further inquiries can be directed to the corresponding author.

## Supplementary material

Supplementary Table 1 Baseline tables of clinical data in the TCGA-LIHC dataset and hepatocellular carcinoma dataset in the ICGC.

Supplementary Table 2 85 pyroptosis gene sets.

Supplementary Table 3 16 immune cell related gene sets.

Supplementary Table 4 13 immune pathway related gene sets.

Supplementary Table 5 The sequences of primers information used in RT-qPCR.

Supplementary Table 6 Differential genes between different cell types.

Supplementary Table 7 Correlation coefficient between PRGs.

Supplementary Table 8 P value of PRGs.

Supplementary Table 9 DEGs between high scores cells.

Supplementary Table 10 GO enrichment analysis of DEGs among high scores cells.

Supplementary Table 11 GO enrichment analysis of 22 intersection genes.

Supplementary Table 12 Univariate COX analysis of risk scores combined with clinical features.

Supplementary Table 13 Multivariate COX analysis of risk scores combined with clinical features.

Supplementary Table 14 Multivariate Cox regression analysis using tumor stages and pyroptosis-related risk scores.

Supplementary Table 15 Correlation between IC50 values and gene expressions.

Supplementary Table 16 The sequences of primers information used in RT-qPCR.
