# Supplementary material for: Prognostic model construction and immune microenvironment analysis of pyroptosis-related genes in hepatocellular carcinoma based on single-cell RNA sequencing
Source: Front Immunol. 2025 Aug 21;16:1595539. doi: 10.3389/fimmu.2025.1595539 (PMC12408283; doi:10.3389/fimmu.2025.1595539)
Supplement: Supplementary Table 1 — Baseline tables of clinical data in the TCGA-LIHC dataset and hepatocellular carcinoma dataset in the ICGC. [file DataSheet1.zip › Data Sheet 1.DOCX]

Table S1 Baseline characteristics of HCC patients in TCGA-LIHC and ICGC databases

|  | TCGA | |  | ICGC | |
| --- | --- | --- | --- | --- | --- |
|  | Lowrisk | Highrisk |  | Lowrisk | Highrisk |
|  | (N=190) | (N=147) |  | (N=135) | (N=96) |
| Age |  |  |  |  |  |
| Mean (SD) | 60.3 (12.6) | 59.5 (14.2) |  | 68.9 (8.36) | 65.1 (11.9) |
| Median [Min, Max] | 62.0 [21.0, 85.0] | 62.0 [17.0, 86.0] |  | 70.0 [46.0, 89.0] | 67.0 [31.0, 85.0] |
| Gender |  |  |  |  |  |
| male | 136 (71.6%) | 94 (63.9%) |  | 106 (78.5%) | 64 (66.7%) |
| female | 54 (28.4%) | 53 (36.1%) |  | 29 (21.5%) | 32 (33.3%) |
| Stage |  |  |  |  |  |
| I | 102 (53.7%) | 66 (44.9%) |  | 24 (17.8%) | 12 (12.5%) |
| II | 46 (24.2%) | 36 (24.5%) |  | 65 (48.1%) | 40 (41.7%) |
| III | 40 (21.1%) | 43 (29.3%) |  | 40 (29.6%) | 31 (32.3%) |
| IV | 2 (1.1%) | 2 (1.4%) |  | 6 (4.4%) | 13 (13.5%) |
| Event |  |  |  |  |  |
| Alive | 139 (73.2%) | 84 (57.1%) |  | 122 (90.4%) | 67 (69.8%) |
| Death | 51 (26.8%) | 63 (42.9%) |  | 13 (9.6%) | 29 (30.2%) |
| Time |  |  |  |  |  |
| Mean (SD) | 30.7 (25.4) | 23.3 (23.2) |  | 28.0 (13.8) | 25.8 (14.1) |
| Median [Min, Max] | 20.8 [0.200, 123] | 14.8 [0.200, 116] |  | 28.0 [0.667, 57.0] | 24.5 [0.333, 72.0] |
| Riskscore |  |  |  |  |  |
| Mean (SD) | -0.296 (0.226) | 0.383 (0.335) |  | 6.00 (0.269) | 6.79 (0.329) |
| Median [Min, Max] | -0.258 [-1.11, 0.00141] | 0.290 [0.00270, 1.66] |  | 6.05 [5.28, 6.39] | 6.67 [6.39, 7.98] |
