# Supplementary material for: Prognostic model construction and immune microenvironment analysis of pyroptosis-related genes in hepatocellular carcinoma based on single-cell RNA sequencing
Source: Front Immunol. 2025 Aug 21;16:1595539. doi: 10.3389/fimmu.2025.1595539 (PMC12408283; doi:10.3389/fimmu.2025.1595539)
Supplement: Supplementary Table 1 — Baseline tables of clinical data in the TCGA-LIHC dataset and hepatocellular carcinoma dataset in the ICGC. [file DataSheet1.zip › Data Sheet 16.DOCX]

Table S16 The sequences of primers information used in RT-qPCR

| **Gene** | **Forward Primer** | **Reverse Primer** | **Product Length**  **(bp)** |
| --- | --- | --- | --- |
| GAPDH | ACAGCCTCAAGATCATCAGC | GGTCATGAGTCCTTCCACGAT | 104 |
| Bax | TCACTGAAGCGACTGATGTCCC | ACTCCCGCCACAAAGATGGTC | 96 |
| CHMP4B | AGAAGCACGGCACCAAAAAC | CGCTGGAACTCGATGGTTGA | 115 |
| CHMP3 | AAGACGGGTTCAGTTCGTCA | GAGCTGGTTCTTCATCCCCA | 243 |
| GBP1 | GAAGATGGAGAACGACAGGGT | ATGGTACATGCCTTTCGTCG | 174 |
| TREM2 | ATGATGCGGGTCTCTACCAG | CTCAGCCCTGGAGATGCTGT | 182 |
| IRF1 | CCAGAGCAGGAACAAGGG | GTGGTCATCAGGCAGAGTG | 196 |
| CHMP2A | TCCGGCCTGCCAGTGA | GAACAGCATCCATCTGGCCT | 206 |
| MST1 | GTGTGCGGGAGAGTGAGATG | TGTGGGTAAAGCAGGCAAGT | 96 |
