# Supplementary figures and images for: Prognostic model construction and immune microenvironment analysis of pyroptosis-related genes in hepatocellular carcinoma based on single-cell RNA sequencing
Source: Front Immunol. 2025 Aug 21;16:1595539. doi: 10.3389/fimmu.2025.1595539 (PMC12408283; doi:10.3389/fimmu.2025.1595539)

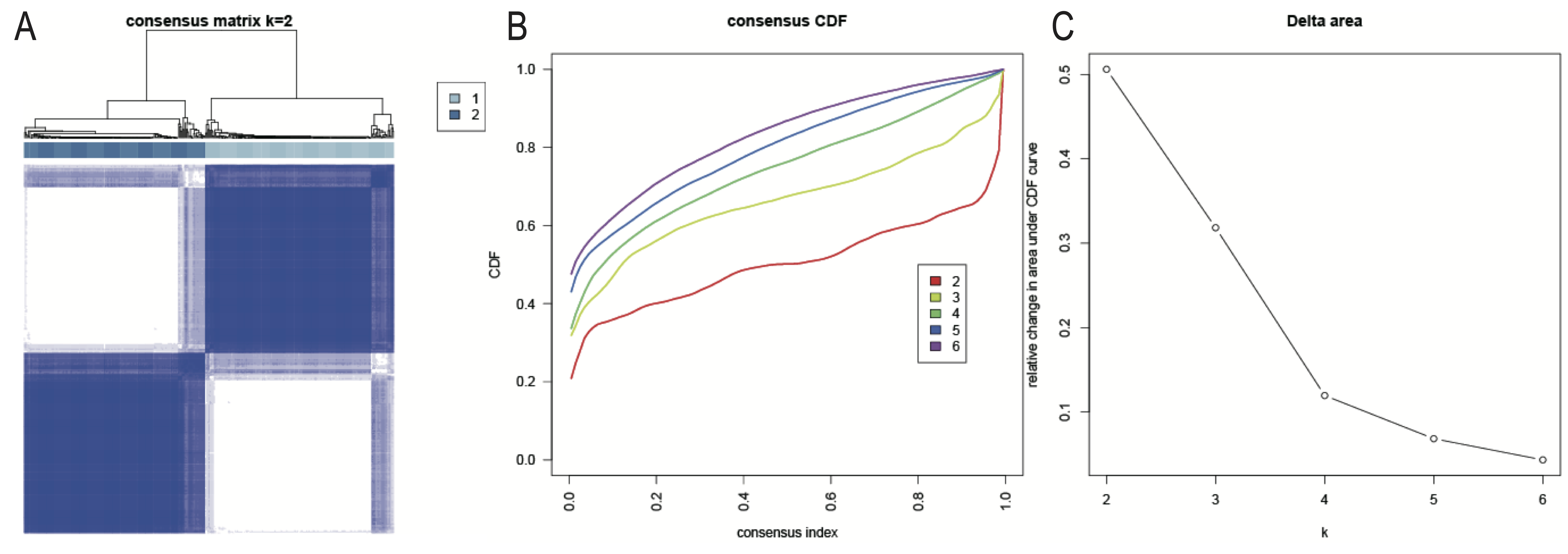

Supplement: Supplementary file 2 [file Image1.tiff]

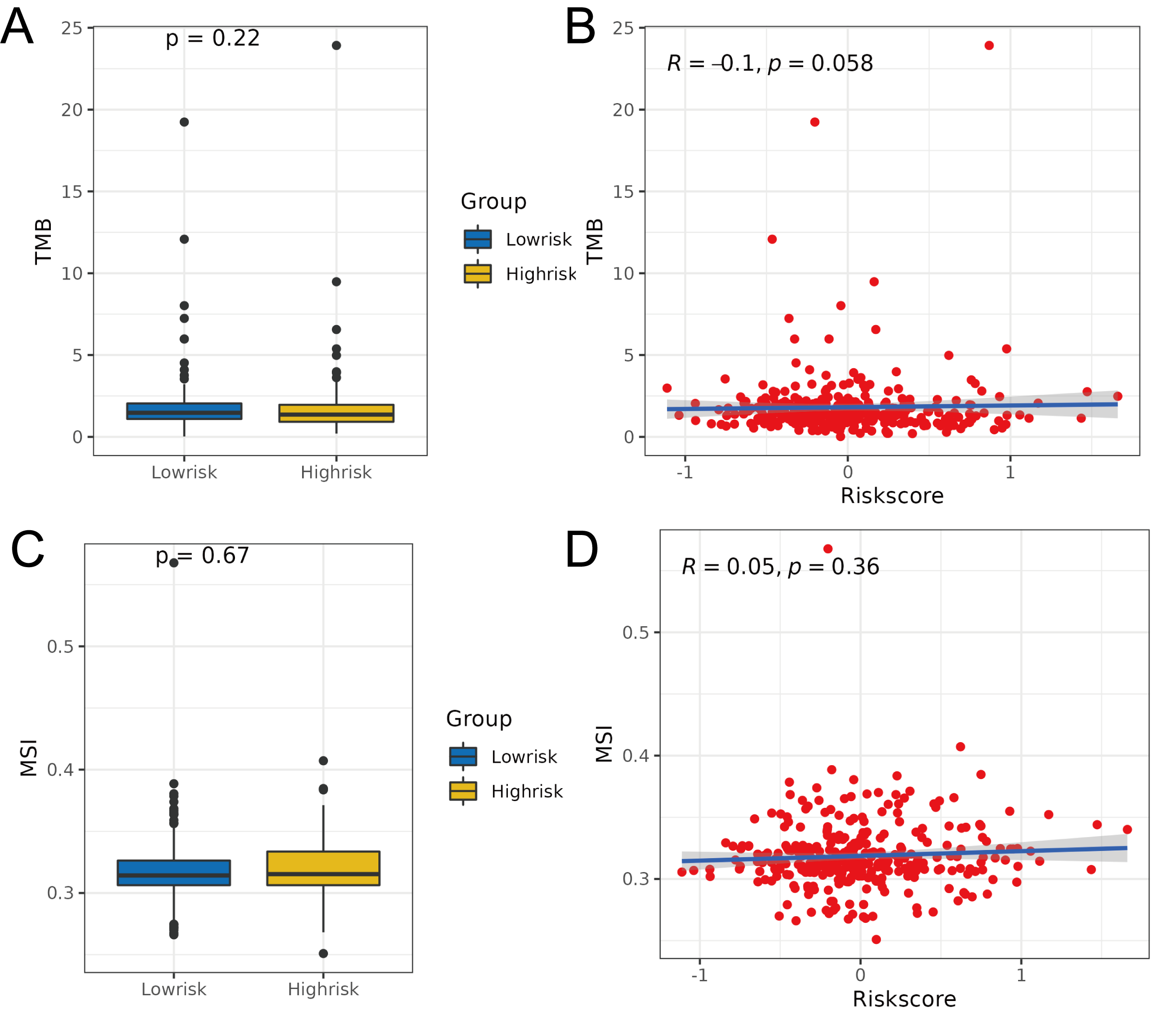

Supplement: Supplementary file 3 [file Image2.tiff]
